# Critical Role for the Human Cytomegalovirus Major Immediate Early Proteins in Recruitment of RNA Polymerase II and H3K27Ac To an Enhancer-Like Element in Ori*Lyt*

**DOI:** 10.1128/spectrum.03144-22

**Published:** 2023-01-16

**Authors:** Eleonora Forte, Ming Li, Fatma Ayaloglu Butun, Qiaolin Hu, Eva Maria Borst, Matthew J. Schipma, Andrea Piunti, Ali Shilatifard, Scott S. Terhune, Michael Abecassis, Jeffery L. Meier, Mary Hummel

**Affiliations:** a Comprehensive Transplant Center, Department of Surgery, Northwestern University Feinberg School of Medicine, Chicago, Illinois, USA; b Proteomics Center of Excellence, Northwestern University, Evanston, Illinois, USA; c Departments of Internal Medicine and Epidemiology, University of Iowa and Iowa City Veterans Affairs Health Care System, Iowa City, Iowa, USA; d Department of Virology, Hannover Medical School, Hannover, Germany; e NUSeq Core, Quantitative Data Science Core, Northwestern University Feinberg School of Medicine, Chicago, Illinois, USA; f Department of Biochemistry and Molecular Genetics, Northwestern University Feinberg School of Medicine, Chicago, Illinois, USA; g Department of Microbiology and Immunology and Biotechnology and Bioengineering Center, Medical College of Wisconsin, Milwaukee, Wisconsin, USA; University of Wisconsin-Madison

**Keywords:** cytomegalovirus, epigenetics, gene expression, herpesviruses, host-pathogen interactions, transcriptional regulation

## Abstract

Human cytomegalovirus (HCMV) is an opportunistic pathogen that infects most of the population. The complex 236 kbp genome encodes more than 170 open reading frames, whose expression is temporally regulated by both viral transcriptional regulators and cellular factors that control chromatin and transcription. Here, we have used state of the art genomic technologies to investigate the viral transcriptome in conjunction with 2 key transcriptional regulators: Pol II and H3K27Ac. Although it is well known that the major immediate early (IE) proteins activate early gene expression through both direct and indirect interactions, and that histone modifications play an important role in regulating viral gene expression, the role of the IE proteins in modulating viral chromatin is not fully understood. To address this question, we have used a virus engineered for conditional expression of the IE proteins combined with RNA and Chromatin immunoprecipitation (ChIP) analyses to assess the role of these proteins in modulating both viral chromatin and gene expression. Our results show that (i) there is an enhancer-like element in Ori*Lyt* that is extraordinarily enriched in H3K27Ac; (ii) in addition to activation of viral gene expression, the IE proteins play a critical role in recruitment of Pol II and H3K27Ac to this element.

**IMPORTANCE** HCMV is an important human pathogen associated with complications in transplant patients and birth defects. The complex program of viral gene expression is regulated by both viral proteins and host factors. Here, we have investigated the role of the immediate early proteins in regulating the viral epigenome. Our results show that the viral immediate early proteins bring about an enormous enrichment of H3K27Ac marks at the OriLyt RNA4.9 promoter, concomitant with an increase in RNA4.9 expression. This epigenetic characteristic adds importantly to the view that OriLyt has structural and functional characteristics of a strong enhancer that, we now discover, is regulated by IE proteins.

## INTRODUCTION

Human cytomegalovirus (HCMV) is a ubiquitous human pathogen of the beta herpesvirus family that latently infects the majority of the population. Primary infection of immunocompetent hosts is typically subclinical, and the infection is kept in check by the host immune response. However, the virus persists in the host indefinitely, and can reactivate under conditions of inflammation or cellular injury (1). In immunocompromised hosts, such as recipients of solid organ or stem cell transplants, reactivation of latent virus can result in significant morbidity or mortality (2). In addition, HCMV infection can be transmitted *in utero* during pregnancy, with potentially devastating consequences (3).

The HCMV genome has the capacity to encode at least 170 open reading frames, 4 major long non-coding RNAs, and multiple microRNAs. The viral genome is not chromatinized in the viral particle, but a proportion of the entering genomes rapidly acquire histones upon entry into the nucleus (4).

Transcription of viral genes is mediated by the host RNA polymerase II and accessory factors, and is enhanced by viral tegument proteins that are transported into the nucleus upon infection, including pp71/UL82, pp65/UL83, ppUL69, pUL28/29, and ppUL35 (5–13). Activation of viral gene expression occurs in a temporally regulated fashion, which has been roughly divided into immediate early, early, early/late, and late phases. Much attention has been focused on activation of the major immediate early (IE) genes, whose transcription is controlled by a strong enhancer element in the major immediate early promoter (MIEP) (reviewed in [14]). Transcripts arising from this locus are differentially spliced to produce two families of proteins, IE1 and IE2. The largest and most abundant of these are the 72 kd IE-1 (IE1-72), and the 86 kd IE-2 (IE2-86) proteins. IE-1 and IE-2 transcripts share the small non-coding exon 1 and coding exons 2 (71 bp) and 3 (185 bp), which encode a nuclear localization signal, but differ in the IE1-specific exon 4 (UL123; 917 bp) and IE2-86-specific exon 5 (UL122; 1487 bp). IE1-72 and IE2-86 are multifunctional regulatory proteins that have major roles in controlling the viral life cycle.

IE1-72 appears to facilitate viral gene expression primarily by antagonizing various host cell defenses. IE1-72 targets both intrinsic immunity through disruption of cellular ND10 structures and innate immunity through disruption of STAT signaling and the interferon response (reviewed in [14]). IE1-72 regulates the viral epigenome by antagonizing de-acetylation of histones bound to the MIEP and by modulating the nucleosome organization of the HCMV genome (15, 16). In addition, IE1-72 tethers the viral genome to cellular chromatin through interaction with an acidic patch on the nucleosome surface formed by histones H2A and H2B, although this tethering is dispensable for viral replication and regulation of viral gene expression (17–20).

IE2-86 is a DNA sequence-specific binding protein, which acts as both a repressor and an activator of viral gene expression (reviewed in [21]). IE2-86 represses its own expression through interaction with IE2-86 binding sites in the *cis* repression sequence (crs) located near the IE transcription start site and recruitment of histone de-acetylases to the MIEP (22, 23). Early studies using reporter constructs showed that it activates early gene expression through IE2-86 binding sites in the promoter regions of some early genes. Multiple cellular transcriptional regulatory proteins have been identified as IE2-86 interaction partners, including AP1, CREB1, EGR1, SP1, p53, Rb, TAF4, TBP, TFIIB, and TFIID, chromatin assembly factor 1 (CAF1), histone acetyltransferases, and histone deacetylases (14, 24–36). Activation of the late genes requires both expression of the late viral transactivators, pUL79, pUL87, pUL91, pUL92, and pUL95, and replication of viral DNA (37–42). Recent genome-wide studies have significantly expanded our understanding of the interactions of IE2-86 with both early and late viral promoters that lead to either repression, activation, or elongation of viral transcripts (43–45).

Among its many binding sites in the viral genome, IE2-86 directly interacts with sites in Ori*Lyt* that are essential for viral DNA replication (43, 45, 46). It is now known that these sites occur in the promoter for the long non-coding RNA4.9. Although its mechanism of action is unknown, recent studies show that this RNA is required for replication of viral DNA (47).

Despite recent advances, the role of the IE proteins in modulating viral chromatin to activate early gene expression remains poorly understood. We previously characterized genome-wide, dynamic changes in the viral transcriptome in conjunction with changes in the epigenome in the Kasumi-3 latency model (48). Here, we have performed similar analyses in the lytic model of fibroblast infection. In addition, we have investigated the role of the IE proteins in regulation of the viral epigenome by constructing a TB40/E HCMV variant in which both the IE1-72 and IE2-86 proteins are conditionally expressed. We found that these proteins have a key role in epigenetic modulation of a vital regulatory region in the HCMV genome.

## RESULTS

### Landscape of the HCMV transcriptome and epigenome during lytic infection.

Here, we have investigated aspects of the HCMV epigenome in the prototypical model of lytic infection. We focused on analyzing genome occupancy of 2 factors with crucial roles in regulating transcription: RNA Polymerase II (Pol II) and H3K27Ac. While RNAseq measures the abundance of mature RNA, which is the net result of transcription, processing, and degradation of the RNA, Chromatin Immunoprecipiration-DNA sequencing (ChIPseq) for Pol II occupancy reflects Pol II’s involvement in pre-initiation, transcription intiation, or productive elongation. Acetylation of histones is important in activation of transcription, and H3K27Ac is localized specifically to enhancer and promoter regions (49, 50). Collectively, these analyses provide greater insight into regulation of transcription than analysis of RNA expression alone.

**FIG 1:**
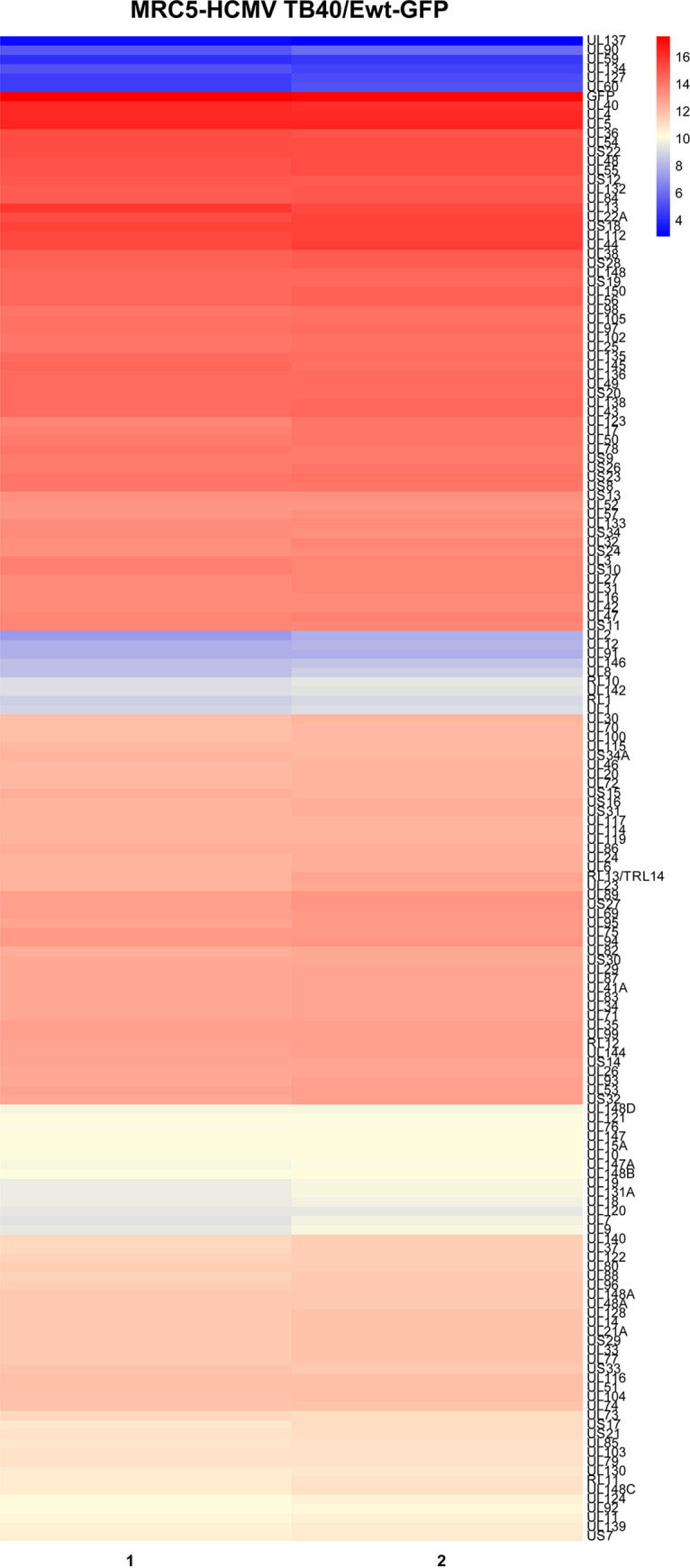
Heatmap of HCMV RNAs expressed in TB40/Ewt-GFP-infected cells at 24 hpi. Data is shown as the log_2_ of reads of 2 independent biological replicates. The genes are clustered on the heatmap by similarity in expression.

**FIG 2:**
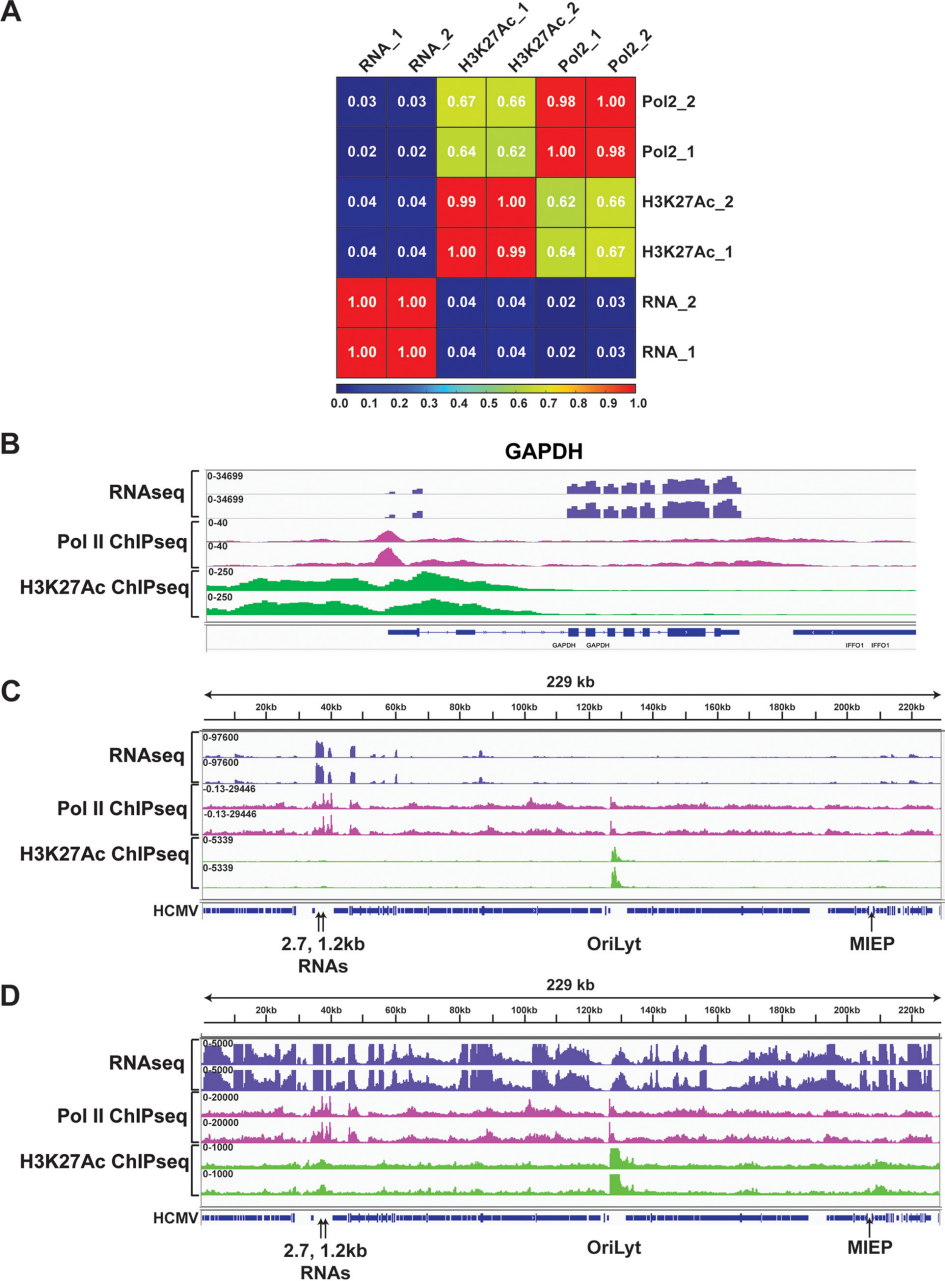
Landscape of the HCMV transcriptome and epigenomes in TB40/Ewt-GFP-infected cells. (A) Pearson correlation analysis between replicates of RNAseq, Pol II ChIPseq, and H3K27Ac ChIPseq in infected MRC5 cells at 24 hpi. (B) RNAseq, Pol II ChIPseq, and H3K27Ac ChIPseq coverage maps for GAPDH in HCMV-infected MRC5 cells at 24 hpi. Tracks shown are from 2 independent experiments. (C and D) Bigwig tracks of two independent replicates of RNAseq, Pol II ChIPseq, and H3K27Ac ChIPseq from TB40/E*wt*-GFP-infected MRC5 cells harvested 24 hpi. Tracks in D are shown at enlarged scale to allow visualization of smaller peaks.

MRC5 fibroblasts were infected at multiplicity of infection (MOI) of 2 and harvested at 24 hours postinfection (hpi) for RNAseq and ChIPseq analyses of Pol II and H3K27Ac occupancy. This time point corresponds to the early phase of infection, when the IE proteins have activated early gene expression and downregulated their own expression, but amplification of viral DNA has not yet occurred. Two independent biological replicates were analyzed. Almost all viral genes were expressed at this time, but there were major differences in the relative abundance of the transcripts (Fig. 1, Fig. S1, and Table S1). As others have previously noted, expression of the 2.7 and 1.2 kb non-coding RNAs was far more abundant than viral transcripts arising from protein-coding regions (Fig. 2) (51–53). ChIP-seq analyses were validated by Pearson analysis, which showed a strong correlation between the replicates (Fig. 2A), and by visual analysis of the tracks for selected cellular genes (e.g., GAPDH), which showed the expected occupancy of Pol II and H3K27 at promoter regions of highly expressed genes (Fig. 2B). ChIP-seq analyses of the viral genome revealed major peaks of Pol II at the promoters for the 1.2, 2.7, and Ori*Lyt* 4.9 kb non-coding RNAs, with many additional smaller peaks throughout the genome (Fig. 2C and Fig. S1). In keeping with the complex array of factors that control RNA expression, there were many differences between the peaks of Pol II occupancy and RNA abundance. Similar to HCMV-infected Kasumi-3 cells, we observed a major peak of H3K27Ac at Ori*Lyt*, which dwarfed all other peaks at this time. However, many smaller peaks of H3K27Ac were detectable throughout the genome when it was visualized at an enlarged scale (Fig. 2D and Fig. S1).

**FIG 3:**
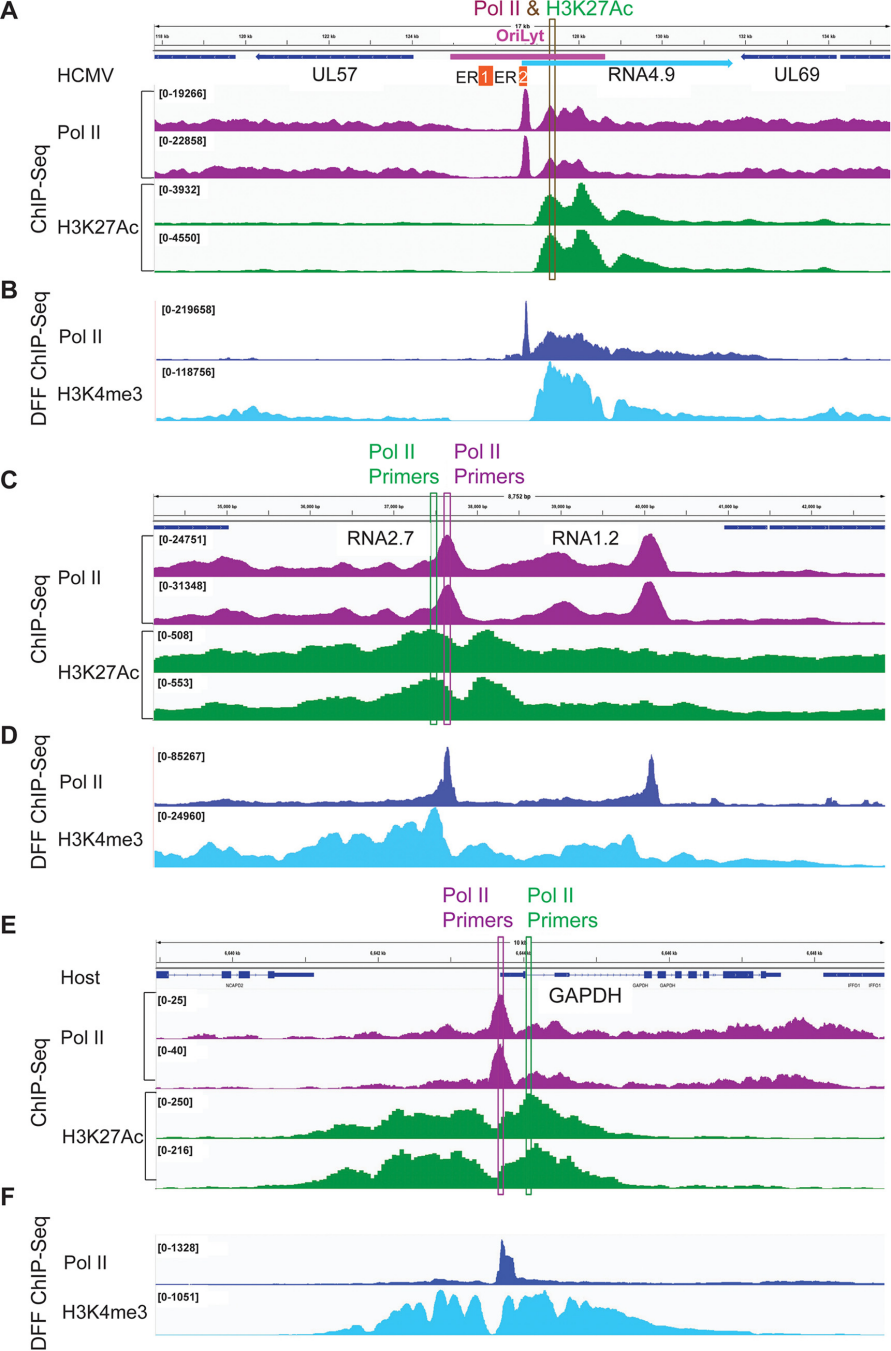
Genome Browser views of position and density of Pol II, H3K4me3, and H3K27Ac signals in promoter proximal regions for HCMV TB40/E Ori*Lyt* RNA4.9 and RNA2.7 and host GAPDH. ChIPseq results at 24 hpi in MRC5 are shown in panels (A, C, and E). DFF ChIPseq results at 48 hpi in HFF are shown in panels (B, D, and F). The annotation in panel (A) depicts location of Ori*Lyt* (pink bar), as well as its essential regions (ER) 1 and 2 in relation to RNA4.9. The location of primers sets used for Pol II and H3K27Ac ChIP-PCR are depicted in panels (A, C, and E).

Spector et al. have recently published data sets allowing a genome-wide analysis of occupancy of Pol II and H3K4me3 in lytically infected fibroblasts at 48 hpi (54). H3K4me3 is another histone modification that marks transcriptionally active host promoters and enhancers. These data sets were generated using DFF ChIP-Seq, in which the chromatin in uncrosslinked cell nuclei was first digested with DNA Fragmentation Factor endonuclease, and then immunoprecipitated. We leveraged this data set to determine the position and density of H3K4me3 signal around RNA2.7 and Ori*Lyt* RNA4.9 promoters. Despite the differences in time after infection (24 hpi in our study vs 48 hpi in the previous analysis) and methodology, the 2 data sets show a remarkable similarity in the location of Pol II peaks (Fig. 3). The close correspondence of Pol II peaks in these studies allowed us to compare results of H3K27Ac and H3K4me3 ChIPseq experiments. The genome browser views show that the amount of both histone modifications at Ori*Lyt* RNA4.9 vastly exceeds that of RNA2.7 (note the differences in the scales in Fig. 3A and B versus Fig. 3C and D). The RNA4.9 promoter is located in the essential region 2 of Ori*Lyt*, and both H3K4me3 and H3K27Ac marks co-localize immediately downstream of the promoter proximal Pol II peak.

**FIG 4:**
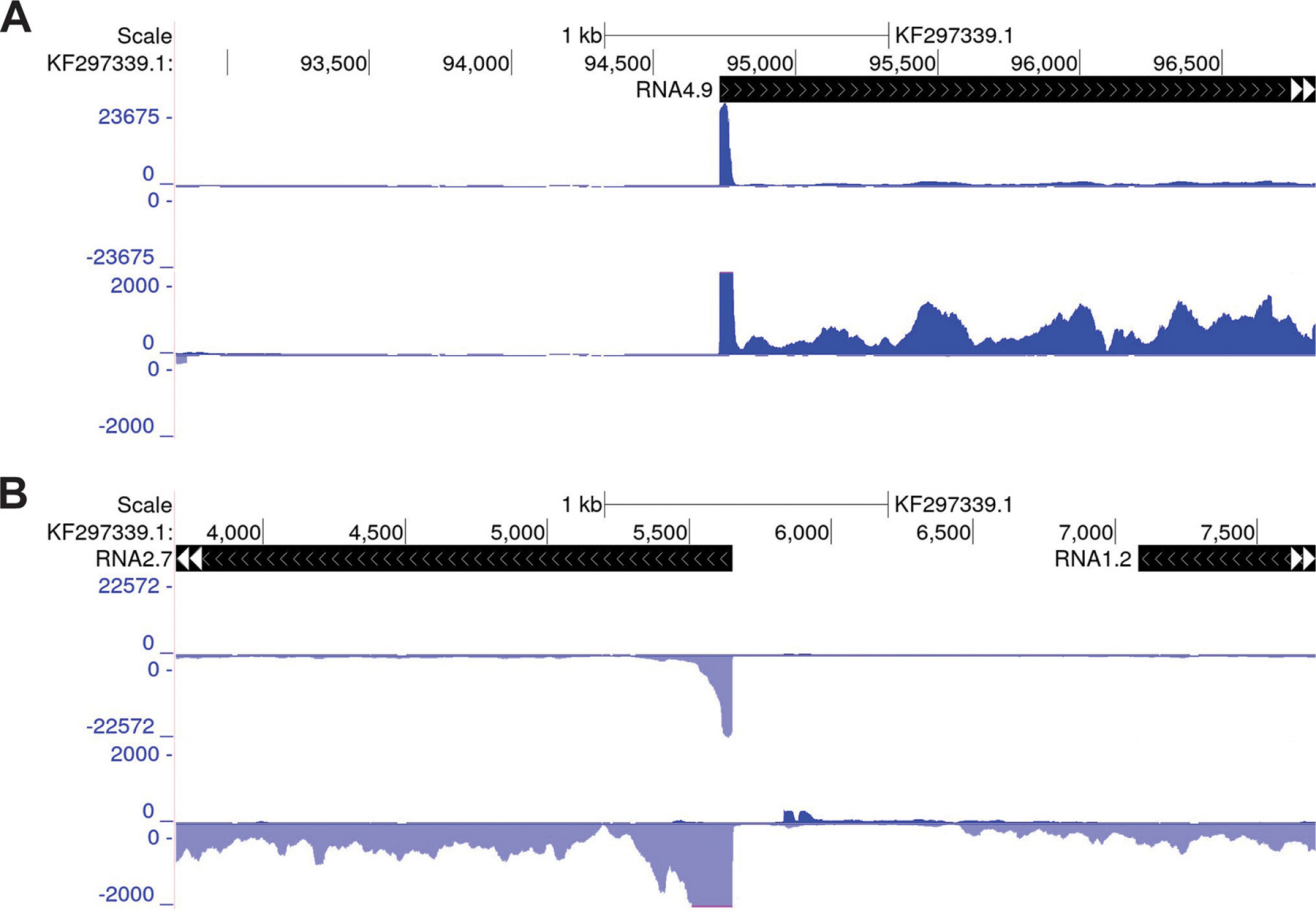
Ori*Lyt* RNA4.9 and RNA2.7 produce a similar amount of Pol II nascent RNA at 24 hpi. HFF infected with TB40/E HCMV (MOI 3) were analyzed by PRO-Seq at 24 hpi. Each aligned read represents a unique Pol II nascent RNA. The UCSC Genome Browser views of Ori*Lyt* RNA4.9 (A) and RNA2.9 (B) were scaled to visualize the amount of promoter proximal paused Pol II (top track) and the amount of Pol II positioned over the gene body (bottom track).

Host enhancers are known to have high content of H3K27Ac nucleosomes. A recent report indicates that histone modifications H3K27Ac and H3K4me3 are dependent on continued transcription by Pol II, as determined by Precision Run-On sequencing (PRO-Seq), which directly measures nascent transcripts (55). The PRO-Seq approach enables nucleotide resolution mapping of position and density of Pol II that is engaged in transcription, regardless of whether the transcripts are stable or not. To determine if the enormous spike in H3K27Ac and H3K4me3 signals at the Ori*Lyt* RNA4.9 promoter was associated with difference in level of nascent transcription, we analyzed PRO-Seq data sets from a 24 h infection in human foreskin fibroblasts (HFFs) that were previously generated by Li et al. (44). At 24 hpi, Ori*Lyt* RNA4.9 and RNA2.7 promoters were the 2 most active of promoters in the viral genome. As shown in Fig. 4, both promoters produced similar amounts of Pol II nascent transcripts positioned in the promoter proximal pause zone and over the gene body. This finding indicates that the enormous difference in H3K27Ac signal at these 2 promoters is not the result of a major difference in level of nascent transcription.

### Construction of TB40r mGFP-IE-FKBP virus.

**FIG 5:**
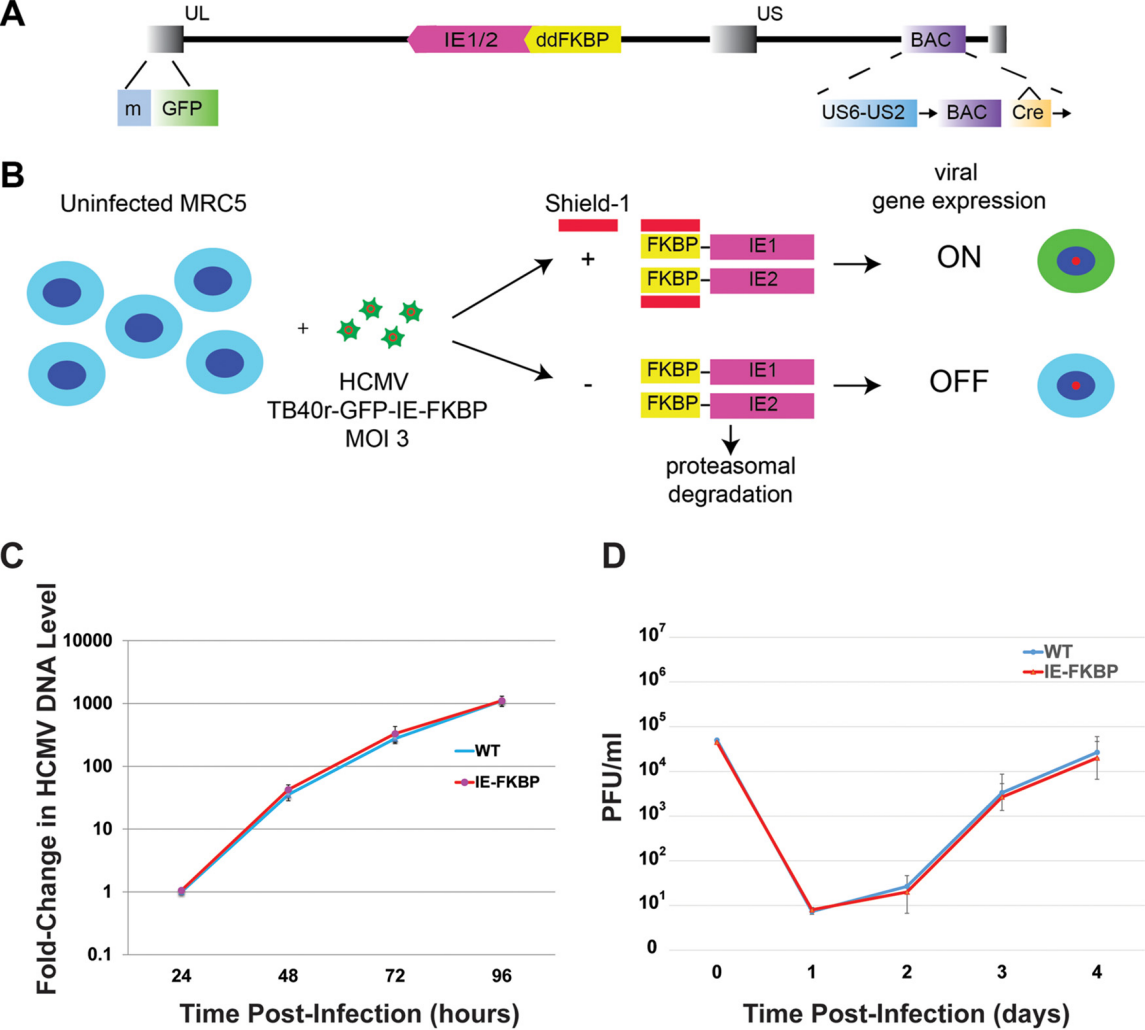
(A) Schematic of the TB40r mGFP-IE-FKBP virus construct used for conditional expression of the IE1 and IE2 proteins. (B) Design of experiments to test the role of the IE proteins in modulating viral gene expression. Shield-1 binds to the FKBP region of the IE fusion proteins to protect them from proteasomal degradation, thus allowing transactivation of early and late genes. (C) Amount of TB40r mGFP-IE-FKBP vs parent WT TB40/E virus genomes produced over time in HFF at MOI 0.5. (D) Amount of infectious TB40r mGFP-IE-FKBP vs parent WT TB40/E virus produced over time in HFF at MOI 1.0.

The role of the major IE proteins in activating the early viral gene expression program is well known. To investigate the role of IE proteins in shaping the viral epigenome, we constructed a virus with conditional expression of the IE proteins. This approach was taken because the IE2-86 protein is essential for viral replication and the creation of viable complementing cell lines has been problematic. This HCMV construction strategy entailed fusing the degradation domain of the FKBP12 protein (ddFKBP) in-frame to the aminoterminus shared by both IE1-72 and IE2-86 (56). The tagged IE1-72F and IE2-86F proteins are protected from degradation by supplementing the culture media with the synthetic ligand, Shield-1, but they are rapidly degraded in the absence of Shield-1 (56). While this strategy performed well in test-of-principle experiments involving the AD169 variant of HCMV, this laboratory HCMV strain lacks several genes functioning in tropism and establishment of latency (57, 58). Therefore, we placed the IE-FKBP fusion in the clinical-like HCMV TB40/E strain (Fig. 5). This entailed use of recently described TB40R-Cre backbone, a TB40-BAC4-based bacterial artificial chromosome (BAC) containing the genome of the HCMV strain TB40/E. TB40R-Cre harbors a re-insertion of the previously deleted US2-US6 genes, as well as 2 loxP sites flanking both the BAC vector and Cre recombinase sequences, thereby resulting in excision of BAC and Cre sequences upon virus reconstitution in cell culture (59). As with the AD169-based virus, the ddFKBP domain was placed at the IE1/IE2 amino terminus. To facilitate visualization of infected cells, the RL13 open reading frame was replaced with a monomeric GFP reporter gene (mGFP) under the control of the MCMV MIEP.

To analyze the effect of these changes on viral replication, we compared cell-associated viral DNA copy and production of infectious virus in HFFs infected with either TB40r mGFP-IE-FKBP or WT TB40/E virus over time. No differences were observed (Fig. 5C and D).

### The FKBP domain is an effective tool for conditional expression of the IE proteins.

**FIG 6:**
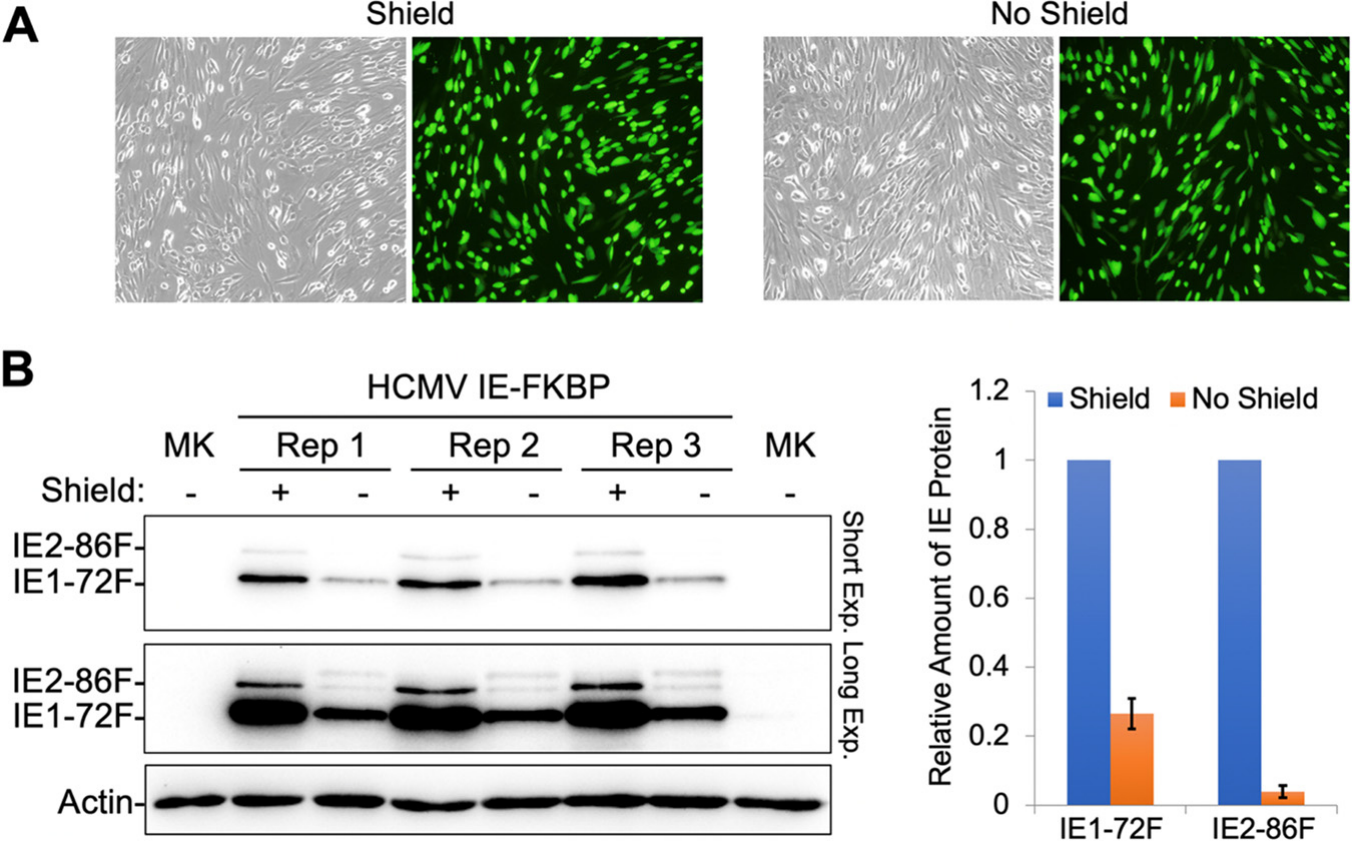
Shield-1 is an effective tool for conditional expression of the IE proteins. (A) Bright field and immunofluorescence images of HFF infected with the FKBP-tagged TB40r mGFP-IE-FKBP virus (MOI 3) with (left) or without (right) addition of Shield-1 throughout the 24 h infection. (B) Left panel, Western blot analysis of expression of IE1-72F and IE2-86F proteins at 24 hpi with (+) or without (-) addition of Shield-1 for 3 independent infections. Actin was analyzed as a normalization control. MK, mock. Right panel, Relative amount of protein expression was quantified using the using the iBright FL1500 Imaging System.

**FIG 7:**
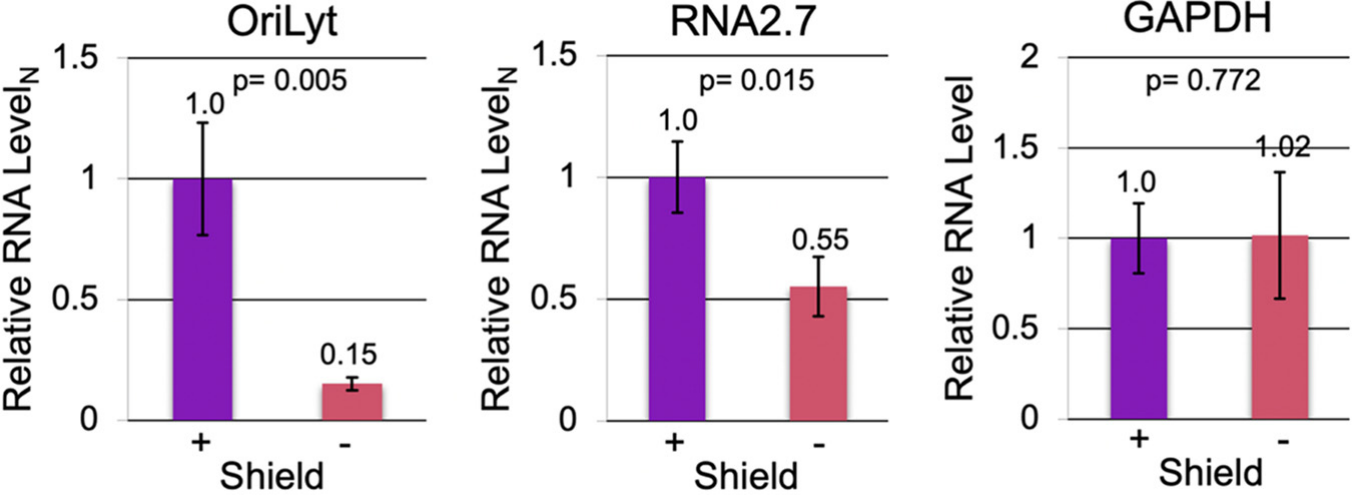
IE protein depletion lowers expression of viral Ori*Lyt* RNA4.9 and RNA2.7. Whole-cell RNA from HFF infected in triplicate with the FKBP-tagged TB40r mGFP-IE-FKBP virus (MOI 3) and treated with (+) or without (-) Shield-1 throughout the 24 h infection was subjected to RT-qPCR. The amount of Ori*Lyt* RNA4.9 and RNA2.7 was normalized to host GAPDH, which did not significantly change when Shield-1 was absent.

To validate conditional expression of the IE proteins conferred by the FKBP domain, we infected HFFs with the TB40r mGFP-IE-FKBP virus in the presence and absence of Shield-1, and analyzed viral gene expression at 24 hpi. No differences were observed in infected cell number, morphology, mGFP protein expression (Fig. 6A). However, expression of both full-length IE1-72 and IE2-86 proteins was significantly reduced in the absence of Shield-1 (Fig. 6B). To test the effect of IE protein depletion on viral RNA expression, we analyzed expression of mature Ori*Lyt* RNA4.9 and RNA2.7 and non-coding RNAs, which are highly expressed in the presence of shield-1. As suspected, depleting the IE proteins by omitting shield-1 decreased levels of RNA2.7 and Ori*Lyt* RNA4.9, without changing host GAPDH RNA level (Fig. 7).

### Depletion of IE proteins significantly reduces recruitment of transcriptional regulators to the Ori*Lyt* RNA4.9 and RNA2.7 promoters.

**FIG 8:**
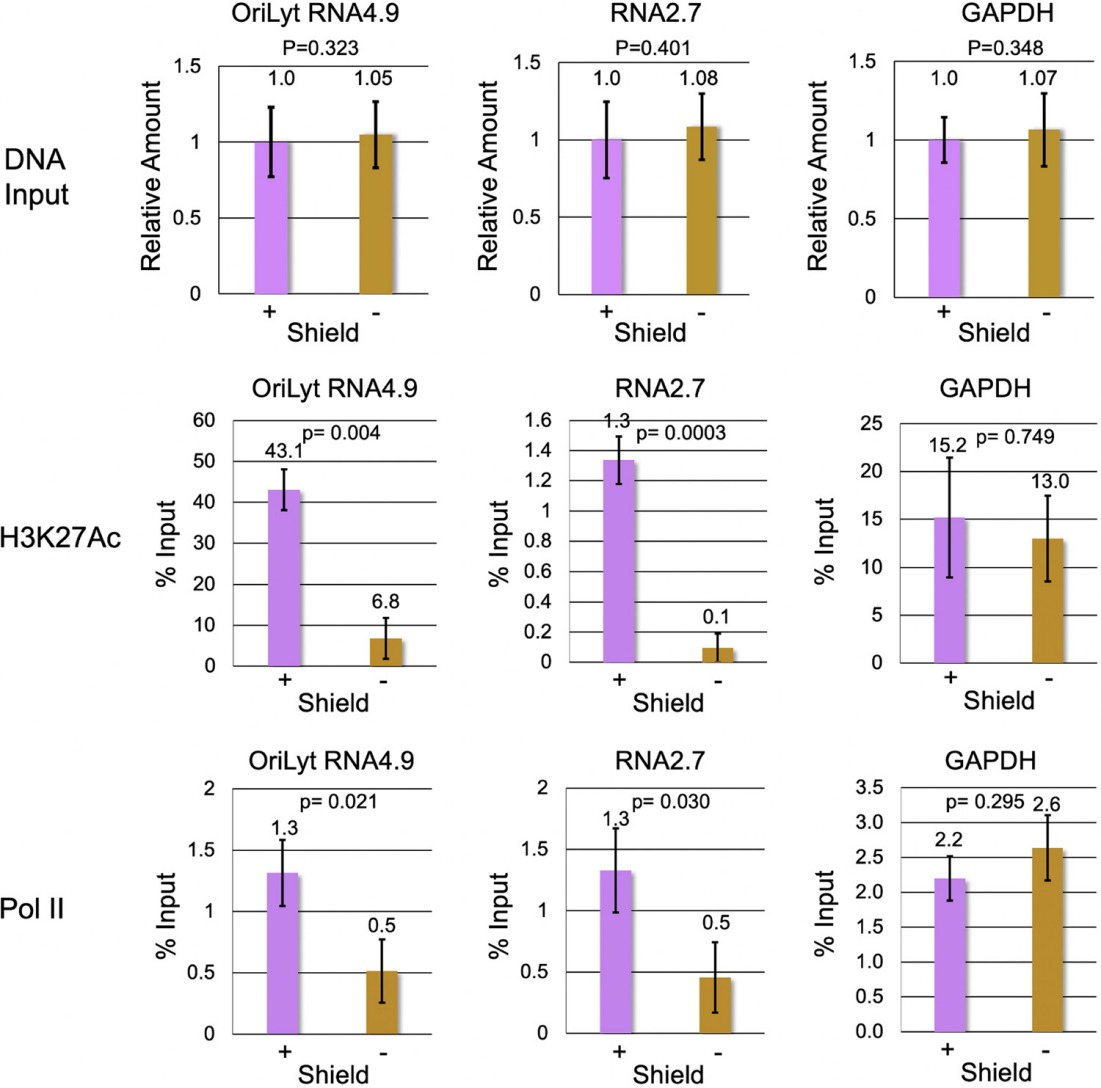
IE protein depletion decreases Pol II and H3K27Ac occupancy of viral promoter proximal gene regions for Ori*Lyt* RNA4.9 and 2.7, but not for host GAPDH. Top panel: qPCR analysis of viral DNA in input cross-linked DNA prior to immunoprecipitation; middle and bottom panels: ChIP-PCR of H3K27Ac (middle) or Pol II (bottom) bound to viral or cellular promoters in cells treated with or without Shield-1. ChIP-qPCR analyses were performed on HFF infected in triplicate with the FKBP-tagged TB40r mGFP-IE-FKBP virus (MOI 3) and treated with (+) or without (-) Shield-1 throughout the 24 h infection. The primer sets depicted in Fig. 3 were used to target the indicated gene regions.

We then examined recruitment of transcriptional regulators Pol II and H3K27Ac, to the RNA2.7 and ori*Lyt* RNA4.9 promoter proximal gene regions in the presence and absence of Shield-1 throughout the 24 h infection. To quantify changes in Pol II and H3K27Ac occupancy, we applied ChIP-qPCR using primer sets that were selected according to Pol II and H3K27Ac ChIPseq peak locations in viral RNA2.7 and Ori*Lyt* RNA4.9 and host GAPDH genes, as depicted in Fig. 3. Viral DNA copy number was analyzed in the input chromatin to verify that equivalent amounts of viral DNA were present under both conditions (Fig. 8, top panel). Our results show that in the presence of Shield-1 the amount of H3K27Ac ChIP-qPCR signal at Ori*Lyt* RNA4.9 is ~ 30-fold greater than that at RNA2.7, which is consistent with the ChIPseq results (Fig. 8, middle panel; note differences in the scales between the 2 genes). In contrast, the amount of Pol II ChIP-qPCR signal is similar for RNA2.7 and Ori*Lyt* RNA4.9, which is consistent with results of both ChIPseq and PRO-Seq (Fig. 8, bottom panel). Omitting shield-1 significantly reduced recruitment of both H3K27Ac and Pol II to RNA2.7 and Ori*Lyt* RNA4.9 promoter proximal gene regions (Fig. 8). This effect was due to depletion of the IE proteins, rather than differences in replication of the viruses, since the samples were analyzed at 24 hpi, prior to the onset of viral DNA replication, and equivalent amounts of viral DNA were present in shield-1-treated and untreated samples. There was no effect on binding to the host GAPDH promoter proximal region, supporting the specificity of the IE protein depletion effect on viral promoters.

IE protein depletion also appeared to decrease H3K27Ac signal at other viral promoters, but this was difficult to measure because of less favorable signal-to-noise ratio. We conclude that the IE proteins activate expression of both RNA2.7 and Ori*Lyt* RNA4.9 in early-stage infection by bolstering the recruitment of Pol II and H3K27Ac, but the OriLyt promoter is more highly enriched in H3K27Ac because it contains an enhancer element.

## DISCUSSION

Like all herpesviruses, expression of genes encoded by the complex HCMV genome is temporally regulated. The initial phase of viral transcription is dominated by the major immediate early genes, whose expression is controlled by the nearby major immediate early promoter/enhancer. Activation of transcription of the early genes requires expression of these proteins, particularly IE2-86 (reviewed in [21]). Although the functional domains of the IE proteins and their interaction partners have been studied extensively, the mechanisms by which they activate early gene expression are not well understood.

Initiation of transcription of cellular genes is a multi-step process involving formation of the Pol II pre-initiation complex at the core promoter region, initiation of short transcripts, pausing of Pol II, and release from pausing (60). Chromatin accessibility, co-activators, and histone modifications all contribute to regulation of these steps. In addition, transcription of many genes is regulated by enhancer regions. Promoters and enhancers were initially classified as distinct entities, in which promoters were defined as the transcription start site and enhancers were defined as elements that amplify initiation. However, recent genomic analyses have revealed that these distinctions are somewhat arbitrary, and that enhancers and promoters share many features (50, 61). It is now understood that enhancer regions are often transcribed bidirectionally, and, like promoters, they are depleted of nucleosomes. In addition, enhancers and promoters both have binding sites for transcription factors that promote CBP/p300-mediated acetylation of H3K27. Although transcription and histone acetylation are highly correlated, the role of histone modifications in activating gene expression has been much debated. One study showed that H3K27Ac is required for transcription of enhancer RNAs and expression of enhancer-proximal genes (62), while another showed that transcription is required for deposition of active histone marks, rather than vice versa (55).

Both enhancers and promoters are marked by mono-, di-, or tri-methylated H3K4 as well as H3K27Ac at the edges of nucleosome depleted regions. However, H3K27Ac is specifically localized to active enhancers and promoters, while H3K4me1 marks all enhancers, whether they are active or not. There are some differences in H3K4 modifications of enhancers and promoters, with active promoters tending to have a higher ratio of H3K4me2/3 to H3K4me1 than enhancers (49). Enhancers of cellular genes may be in close proximity to promoters, but often are located far from the transcription start site. Long-range interactions between promoters and enhancers are facilitated by looping-promoting factors, such as cohesins and long ncRNAs (61, 63).

Although many of these factors also regulate HCMV gene expression, there may be some aspects that are unique to the viral genome. Because viral DNA is not associated with histones in the virion, and nucleosome occupancy remains low during the early stages of infection, nucleosome remodeling to allow the transcription apparatus access to early promoters may have a less prominent role in regulation of viral gene expression (4, 16, 48). Although some genes, including US3, UL36, and UL37, are expressed in addition to IE1-72 and IE2-86 during the immediate early phase, only 1 enhancer, the MIEP, has been previously identified in the HCMV genome. Unlike many cellular enhancers, the MIEP is in close proximity to the genes that it regulates, IE1-72 and IE2-86. The MIEP has binding sites for both activating and repressive transcription factors (reviewed in [14]). Some of these activators, such as NF-κB, ATF/CREB, and SP1, have been shown to be activated by infection (32, 64–66). The specific factors that regulate the MIEP may vary under different circumstances, and therefore may be dependent on the cellular environment (reviewed in [1 and 14]).

Previous studies have also shown that that histones bound to the MIEP are acetylated, and that acetylation of histones is important in activating viral gene expression (15, 23, 67–70). Our recent studies show that the MIEP is also highly H3K27 acetylated during the immediate early phase of transcription in the Kasumi-3 model of latent infection (48).

### Ori*Lyt* is highly enriched with H3K27Ac during the early phase of lytic infection.

In our previous analysis of the viral epigenome in Kasumi-3 cells (48), we showed that there was a transition in the pattern of occupancy of H3K27Ac from the MIEP to Ori*Lyt* that coincided with activation of early gene expression. Like the peak present at the MIEP during the immediate early phase, the peak at Ori*Lyt* dwarfed all other peaks observed at that time. Here, we have investigated genome-wide occupancy of H3K27Ac in a lytic infection model. While many peaks were detected that may have a role in regulating expression of individual genes, we show that H3K27Ac is strikingly enriched at Ori*Lyt* over all other regions at 24 hpi. This peak was localized near the Pol II peaks that were observed upstream of the non-coding RNA4.9 in both our study and those of Parida, et al. (71). We did not normalize H3K27Ac to total H3, and thus, we cannot rule out the possibility that the peak at Ori*Lyt* was due to a skewed distribution of H3. However, our analysis of H3 occupancy of the HCMV genome in Kasumi-3 cells showed that H3 was uniformly distributed across the genome (48). Furthermore, Spector, et al. have recently demonstrated coverage of H3K4me3 over the entire HCMV genome at 48 hpi (54). We therefore favor the possibility that H3K27Ac is specifically recruited to Ori*Lyt* through factors that bind to sequences in this element, rather than enrichment of nucleosomes to Ori*Lyt.* A striking and unique feature of Ori*Lyt* is the presence of multiple G-rich sequences with the potential to form G-quadruplexes (71). Although initially thought to be antagonists of transcription, more recent genomic studies show that these structures are strongly associated with acetylated histones and active transcription (72).

Recruitment of both Pol II and H3K27Ac to the RNA4.9 promoter was dependent on stable expression of the IE proteins. Our studies do not allow us to distinguish the roles of IE1-72 and IE2-86, and both may contribute to this process through alternative mechanisms (15, 23). Our studies raise the intriguing hypothesis that direct or indirect interactions between the IE proteins and G-quadruplexes may be involved in recruiting Pol II and H3K27Ac to the RNA4.9 promoter and activating transcription of this gene.

### Ori*Lyt* contains many of the features of an enhancer.

Ori*Lyt* is a complex region that contains the origin of viral DNA replication. The replication function of Ori*Lyt* is dependent on its position in the HCMV genome, as mutant genomes with an inversion of the 1.5 kbp core origin were defective in replication (73). Two essential regions, essential region I (ERI) and essential region II (ERII), have been identified within Ori*Lyt* (reviewed in [46]). In addition to binding sites for the cellular transcription factor C/EBPα/β, ERI has a crs-like element that binds IE2-86 and has bidirectional promoter activity that is IE2-86- and pUL84-dependent (74). ERII encodes two anti-sense RNAs that form RNA/DNA stem-loop structures that interact with pUL84 (75, 76). More recent studies show that ERII contains the promoter for RNA4.9, which regulates viral DNA replication (47, 71). Targeting of this element in the viral genome with CRISPRi resulted in reduced expression of the single-stranded DNA binding protein UL57 as well as RNA4.9 (47). The UL57 promoter is located on the opposite strand of the DNA 2.8 kb upstream of RNA4.9. This observation suggests that the 4.9 kb promoter proximal sequences can act at a distance to regulate expression of other genes.

Thus, although it was not previously designated as such, accumulating evidence now suggests that Ori*Lyt* encodes a second enhancer-like element that is activated in the early phase of viral gene expression. Previous studies showed that, like classical enhancer elements, Ori*Lyt* encodes a strong promoter with bidirectional activity in reporter assays, and this promoter can drive expression of heterologous genes independently of its orientation (74). Recent studies showed that this element affects not only expression of the downstream RNA4.9, but also the upstream UL57 gene when targeted in the viral genome (47). Analyses of the cellular epigenome have demonstrated a strong correlation between enhancers and the presence of H3K27Ac (49, 50). In this study, we show that, although nascent transcription is equivalent between the RNA2.7 and RNA4.9 promoters, OriLyt is highly and specifically marked with H3K27Ac. We further show that deposition of this mark is dependent on the activity of the IE proteins.

It has long been known that transcription plays an important role in replication of HCMV DNA, as well as that of gamma herpesviruses. This connection has recently been strengthened by the observation that expression of RNA4.9 encoded by the Ori*Lyt* region regulates viral DNA replication (47). Because acetylation of H3K27 is strongly associated with active transcription, our studies suggest that chromatin modifications at Ori*Lyt* link these 2 processes by facilitating expression of RNA4.9.

### Complexity of early gene expression.

The early genes have been broadly defined as those that are dependent on expression of the IE genes, but independent of viral DNA replication. However, within this group, there are 3 subclasses: (i) those that are transcribed early and repressed after the onset of DNA replication; (ii) those expressed at constant levels throughout the replication cycle; (iii) those that are transcribed at low levels early, and subsequently at higher levels. Thus, regulation of early gene expression is likely subject to multiple layers of regulation (77). Recent genome-wide studies have revealed that many viral genes are either up- or downregulated by IE-2 (43, 44). Binding of cellular transcription factors to viral promoters, such as the ATF-1 sites upstream of UL112 and UL54 (78), also likely enhances expression of some genes. In addition, IE2-86 may act indirectly through interaction with other sequence-specific DNA binding factors to activate early gene expression (21, 79).

Our studies show that there are dramatic differences in the landscape of histone acetylation throughout the viral genome. Interestingly, while the highest levels of H3K27Ac were observed at Ori*Lyt*, RNA2.7 and RNA1.2 had the highest levels of Pol II binding and RNA expression. Differences in RNA stability may be a factor in determining the relative abundance of these transcripts. Further studies are needed to unravel the complex layers of regulation of early gene expression, the roles of the individual immediate early proteins in this process, and to identify additional *cis-* and *trans*-acting factors that regulate H3K27 acetylation of Ori*Lyt* and early gene expression.

### Acetylation of H3K27 may be a therapeutic target.

Acetylation of H3K27 is mediated by histone acetyl transferases (HATs) P300 and cyclic AMP response element binding protein (CBP) (80), and this modification is required for enhancer function (62). H3K27Ac is recognized by the bromodomain of BET family members, such as BRD4, and by AFF4, a scaffolding protein that mediates recruitment of the super-elongation complex (SEC) (60, 81). The key catalytic component of both BRD4 complexes and the SEC is positive transcription elongation factor b (pTEFb), which phosphorylates both Ser2 of paused RNA Pol II and the negative regulators DSIF and NELF (60). This promotes release of paused Pol II and productive elongation of transcripts. Recent studies have highlighted the significance of transcriptional pausing and release in regulation of HCMV transcription (40, 71, 82–84). Thus, acetylation of H3K27 at the MIEP and Ori*Lyt* is likely to have a major role in activation of both the immediately early and early phases of HCMV gene expression, respectively.

Small molecule inhibitors targeting the bromodomains of P300 and CBP that specifically block acetylation of H3K27 and transcription of some cellular genes have been identified (62). Current antiviral therapies are directed at inhibiting viral DNA replication via polymerase inhibitors or assembly of viral particles. Use of these compounds is complicated by toxicity and the development of resistant strains. Our studies raise the possibility that specific inhibitors of H3K27 acetylation at Ori*Lyt* could be used as a novel therapeutic approach to block both HCMV gene expression and DNA replication.

## MATERIALS AND METHODS

### Antibodies and reagents.

HCMV IE1/IE2 and pp65-specific antibodies were from Virusys corporation (P1215 and CA003-100, respectively). Murine monoclonal antibody MAB810 against both HCMV IE1-72 and IE2-86 was purchased from EMD Millipore. Rpb1 NTD (D8L4Y) Rabbit MAb #14958 (Pol II) and Acetyl-Histone H3 (Lys27) (D5E4) XP Rabbit MAb #8173 were purchased from Cell Signaling Technologies. Anti-Pol II antibody F-12 (sc-55492) used for ChIP-PCR studies was from Santa Cruz. Polyclonal rabbit anti-actin antibody used for Western blots (A2066) was from Sigma-Aldrich. Shield-1 was obtained from Clontech (632188) and diluted to 5 mg/mL in Ethanol. Protein A/G plus-Agarose was from Santa Cruz Biotechnology (sc-2003), whereas Pierce Protein A/G Magnetic Beads were from Thermofisher (88802). Oligonucleotides were produced by Integrated DNA Technologies.

### Cell culture.

HFFs were derived from discarded deidentified human foreskins in accordance with University of Iowa IRB number 201702743. MRC5 cells were obtained from ATCC (CCL-171). Cells were cultured in Dulbecco’s Modified Eagle Medium (DMEM) medium (Gibco) supplemented with 10% fetal bovine serum (FBS) (Corning) and 1,000 Units/mL Penicillin-Streptomycin (Gibco) at 37°C in a humidified incubator at 5% CO_2_. Cells infected with TB40r mGFP-IE-FKBP were kept in the presence of 1 μM Shield-1 (+Shield) or Ethanol (-Shield).

### Construction of TB40r mGFP-IE-FKBP virus.

The HCMV mutant generated in this study is based on the recently described TB40R-Cre backbone, a TB40-BAC4-based bacterial artificial chromosome (BAC) containing the genome of the HCMV strain TB40/E. TB40R-Cre harbors a re-insertion of the previously deleted US2-US6 genes, as well as 2 loxP sites flanking both the BAC vector and Cre recombinase sequences, thereby resulting in excision of BAC and Cre sequences upon virus reconstitution in cell culture (59). To replace the RL13 open reading frame (ORF) of TB40R-Cre by sequences encoding the monomeric EGFP (mGFP) under the control of the MCMV major immediate early promoter via *en passant* mutagenesis (85), plasmid pMCMV-mGFP-in was constructed by Gibson assembly following the manufacturer’s instructions (Gibson assembly master mix, cat. no. E2611S, New England BioLabs). The mGFP ORF comprising an internal KnR cassette (plus elements required for *en passant* mutagenesis) was PCR-amplified from pEP-mGFP-in (86) using primers mGFP-in.for (5′-CGCTGCAGCCCGGGTATGGTGAGCAAGGGCGAG-3′) and mGFP-in.rev (5′-ACGTCGACGGATCCTTTACTTGTACAGCTCGTCCATGC-3′) and cloned into plasmid pMCMV3 (87) cut with XbaI. A PCR fragment was then produced from pMCMV-mGFP-in using primers TB40-RL13-MIEP.for (5′-ACAACATCCGAAGAAACATCAATGCCCATTAACCGAAATCCAACAACGTTTAAACGGTACTTTCCCATAGCTG-3′) and TB40R-RL13-mGFP.rev (5′-GAAACATATTATTGGCTAAAAAGAAAAGCAAAAGTTTATTGGTGTGCATGTTACTTGTACAGCTCGTCCAT-3′) and recombined with the TB40R-Cre BAC genome followed by excision of the KnR marker, giving rise to TB40R-RL13-mGFP. For tagging of the IE1/IE2 exon 2 locus in TB40R-RL13-mGFP with the ddFKBP sequences by traceless *en passant* mutagenesis a PCR template comprising ddFKBP with an internal KnR cassette was constructed. The KnR resistance gene together with features needed for *en passant* mutagenesis was obtained by PCR from pori6K-RIT (59) as template and primers FKBP-Kan-in.for (5′-CTGACCCACACTCATCTGGGCAACCCCTTCTTCCCAGCCTCGGATCACCTCCTGCTTGCCGACGCATCGTGGCCGGATC-3′) and FKBP-Kan-in.rev (5′-AGCCCTTTAAGTTTATGCTAGGCAAGCAGGAGGTGATCCGAGGCTGGGAAGAAGGGGTTGGTGACCACGTCGTGGAATG-3′), and subsequently inserted into the ddFKBP locus of the previously described AD169-IE1/IE2-ddFKBP BAC (56) by homologous recombination in E. coli. The resulting genome, AD169-IE-FKBP-Kn was then used to generate a PCR product encompassing ddFKBP-Kn for mutagenesis of TB40R-RL13-mGFP, employing primers IE-FKBP.for (5′-CAGGGTTGTCAGGGTCCATCTTTCTCTTGGCAGAGGACTCCAATTGGCGCGCGGATCCT-3′) and IE-FKBP.rev (5′-TCCATGGGTCTTTTCTGCAGTCACCGTCCTTGACACGATGGGAGTGCAGGTGGAAACCA-3′). Excision of the KnR marker finally yielded TB40R-mGFP-IE-FKBP. All constructs generated were verified by restriction analysis and sequencing of the relevant regions.

### Virus stocks.

TB40/E*wt*-GFP (88) and TB40r mGFP-IE-FKBP viral stocks were produced as previously described (89), with the difference that for the mutant virus we supplemented the culture media with 1 μM Shield-1 dissolved in ethanol to allow stabilization of IE1/2 proteins, or with ethanol (vehicle) only. Titers of the viral stocks were determined on MRC-5 using the 50% tissue culture infective dose (TCID_50_) method.

### Immunoblotting.

Western blot was performed on triplicate infections carried out in confluent HFF in 6-well plates at the indicated MOI and treatment conditions. Sonicated whole-cell extracts in lysis buffer containing phosphatase and protease inhibitors were prepared as previously described (44). The gel-loading buffer contained 2% SDS and 100 mM beta-mercaptoethanol. The proteins were fractionated on freshly made 8% SDS-PAGE Tris-glycine gels prior to transfer to Amersham Protran 0.45-μm nitrocellulose membranes (GE Healthcare Life Sciences 10600002). HCMV IE1-72 and IE2-86 were detected by murine monoclonal antibody MAB810 (EMD Millipore, 1:1,000 dilution). Host actin was detected with polyclonal rabbit anti-actin antibody (Sigma-Aldrich, A2066, 1:4,000 dilution). Primary antibodies were detected with peroxidase AffiniPure F(ab’)2 fragment goat anti mouse IgG (Jackson ImmunoResearch, 115-036-006) at 1:40,000 dilution and goat anti-rabbit IgG (whole molecule)–peroxidase antibody (Sigma-Aldrich, A0545) at 1:40,000 dilution. Blots were imaged and analyzed using the iBright FL1500 Imaging System (Invitrogen).

### MRC5 infection.

For each infection, 5 × 10^6^ MRC5 were seeded in 15 cm dishes the day before the infection. The following day, medium was replaced with 10 mL of DMEM supplemented with 10% FBS without antibiotics. Confluent MRC5 were infected with HCMV at a MOI of 2 with TB40/E*wt*-GFP. After 1 h incubation at 37°C, 10 mL of media without antibiotics was added to the plate and the cells incubated at 37°C for 23 h. Mock-infected cells were prepared in parallel to infected cells, replacing the virus with the same amount of phospahte-buffered saline (PBS). The infection with TB40R-mGFP-IE-FKBP mutant was carried out in the presence of 1 μM Shield-1 (+Shield) or Ethanol (-Shield) at MOI of 3. The infection was conducted as described above for TB40/E*wt*-GFP. Cells were collected at 1 dpi.

### RNA and DNA extraction.

For extraction of RNA used in RNA-Seq, cells were lysed in TRIzol and total RNA was prepared using Direct-zol RNA miniprep kit (Zymo Research) following the manufacturer’s instructions that include an on-column DNase digestion step. DNA was prepared with EZ1 DNA Tissue Kit on a EZ1 instrument (Qiagen), as instructed by the manufacturer.

### Real-time PCR analysis.

Whole-cell RNA was isolated using the TRIzol Reagent (Invitrogen, 15596026) method and reverse transcription (RT) was performed using SuperScript III Reverse Transcriptase (Invitrogen, 18080093), according to the manufacturer’s instructions. The cDNA target was quantified by real-time PCR (qPCR) using the Applied Biosystems 7500 Fast real-time PCR system. PCR was performed using oligonucleotide primers listed in Table 1 that detect RNA4.9, RNA2.7, and glyceraldehyde-3-phosphate dehydrogenase (GAPDH) cDNA. This was carried out in Power SYBR green PCR Master Mix (Thermo Fisher, 4367659) using the thermocycling parameters of 95°C for 10 min, 40 cycles at 95°C for 15 s, and 60°C for 60 s. The amount of each specific viral cDNA target was determined by standard curve method and normalized to amount of host GAPDH cDNA target. Triplicate infections for each treatment condition were carried out in parallel in 12-well plates.

**TABLE 1:** Oligonucleotides

Oligo name	Direction	Sequence (5′-to-3′)
RT-qPCR Oligos		
OriLyt	Forward	GTAAGACGGGCAAATACGGT
	Reverse	AGAGAACGATGGAGGACGAC
RNA2.7	Forward	TCTCTTCTCTCTCTACATACAGACC
	Reverse	CCATAATCATCGAAGAATGAAAGACG
GAPDH	Forward	CTGTTGCTGTAGCCAAATTCGT
	Reverse	ACCCACTCCTCCACCTTTGAC
ChIP-qPCR Oligos		
OriLyt Pol II and H3K27Ac	Forward	GACGGCTTCCGGGTCT
	Reverse	GCCGGACCCTCGAGAG
RNA2.7 Pol II	Forward	GAGCAGCAGCGATCTGG
	Reverse	CACACGTCTTTCCGCTTACT
RNA2.7 H3K27Ac	Forward	CGAGATTCGACCAGACAGAAG
	Reverse	GGAGCCGAGATGACAACAG
GAPDH Pol II	Forward	CTCCCGCTTCGCTCTCT
	Reverse	TTTCTCTCCGCCCGTCTT
GAPDH H3K27Ac	Forward	CAGTCAGCCGCATCTTCTTT
	Reverse	CCTTCAGGCCGTCCCTA

### RNAseq and data analysis.

RNA quality was assessed on a Bioanalyzer, and libraries were prepared from 2 independent experiments using Illumina Ribo-zero (TB40/E wt gfp-infected cells) kits according the to manufacturer’s instructions. Libraries were sequenced on an Illumina NovaSEQ6000 (100 bp paired-end reads). The quality of DNA reads, in FASTQ format, was evaluated using FastQC. Reads were trimmed to remove Illumina adapters from the 3′ ends using a cutadapt wrapper, Trimgalore (90, 91). Trimmed reads were aligned to the human genome (hg19) or HCMV TB40/E clone TB40-BAC4 (EF999921.1), to which the GFP gene sequence was added (available at https://github.com/Mary-Hummel/HCMV-reference-genomes) using STAR (92). STAR was also used to calculate the read counts/gene through the -quantMode gene counts option. Heatmaps were generated from the log_2_ of the raw read counts using the R function of pheatmap.

### ChIP-seq and data analysis.

Chromatin was prepared from cells fixed with 1% formaldehyde as previously described (48). Libraries were prepared with the Illumina Chip-SEQ Kit multiplexed with IDT unique dual index barcodes. Library quality control was assessed using Bioanalyzer High Sensitivity DNA Analysis kit (Agilent) and sequenced on an Illumina NovaSEQ6000 (100 bp paired-end reads). The quality of reads, in FASTQ format, was evaluated using FastQC. Reads were trimmed to remove Illumina adapters from the 3′ ends using cutadapt (93). Trimmed reads were aligned using Bowtie2 (version 2.2.9) with default parameters to the human genome (hg19) and the HCMV TB40/E clone TB40-BAC4 sequence (EF999921.1), to which the GFP gene sequence was added. Reads mapping uniquely to the genomes were further analyzed with Hypergeometric Optimization of Motif EnRichment (HOMER, version 4.11) (94) to call Pol II and H3K27Ac peaks, and generate IGV read density tracks.

UCSC Genome Browser views of selected Pol II and H3K4me3 DFF ChIP-Seq results for HCMV TB40/E-infected fibroblasts at 48 hpi were generated from Pol II (GSM5620717) and H3K4me3 (GSM5620716) bigwig files that are publicly available at GEO (GSE185763). UCSC Genome Browser views of PRO-Seq results for regions around Ori*Lyt* RNA4.9 and RNA2.7 promoters were generated from bigwig files that are publicly available at GEO (GSE GSE139114).

### Viral growth curve and DNA replication assay.

The TB40r WT and IE-FKBP viral stocks were generated in parallel and titered on HFF by application of immunofluorescence assay of IE1/IE2 protein-positive cells at 24 hpi. This assay entailed use of murine monoclonal antibody MAB810 (EMD Millipore, 1:1000 dilution) and secondary goat anti mouse IgG (H+L) antibody conjugated to Alexa Fluor 555 (Thermo Fisher Scientific, A-21422). Low passage-number HFF (≤ 6) were grown in Minimum Essential Medium (Gibco, 11095080) supplemented with heat-inactivated 8% FBS (Gibco, 26140079) and 1000 U/mL penicillin-streptomycin (Gibco, 15140122). The viral growth curve and DNA replication assay were performed in parallel. HFF were infected in triplicate with TB40r WT and IE-FKBP at the indicated MOI, and the infected cells were washed thrice after the 90-min viral adsorption. For the viral growth curve, the infected cells were scraped into medium at each of the designated time points and stored at −80°C. The set of samples was then thawed, sonicated (10 min), centrifuged (1,000 × *g* for 10 min), supernatants passed through a 0.45 μm filter, and amount of infectious virus in the supernatant was determined by standard plaque assay performed on subconfluent HFF cells. For viral DNA replication assay, the infected cells were washed in 1x PBS and resuspended in ice-cold buffer A (10 mM HEPES [pH 8.0], 1.5 mM MgCl_2_, 10 mM KCl, 1 mM dithiothreitol) containing 0.1% Triton X-100. Pipetting up and down ensured pellet is resuspended completely. Pelleted nuclei were resuspended in PCR lysis buffer (10 mM Tris-HCl [pH 8.0], 1 mM EDTA, 0.001% Triton X-100, 0.0001% SDS) containing 20 μg/mL proteinase K. The lysate was incubated at 55°C for 100 min and the proteinase K was then heat-inactivated at 95°C for 20 min. Viral DNA was quantified by real-time PCR using oligonucleotide primers targeting viral IE1 exon 4 as previously described (44) and Power SYBR green PCR Master Mix (Thermo Fisher, 4367659). The 7500 Fast real-time PCR system (Thermo Fisher) was used with parameters of 95°C for 10 min, followed by 40 cycles at 95°C for 15 s, then 60°C for 60 s. Results were normalized to host GAPDH DNA.

### ChIP-qPCR.

Confluent HFF infected with TB40R-mGFP-IE-FKBP virus at MOI of 3 in T75 flasks were treated with 1 μM Shield-1 (+Shield) or ethanol vehicle (-Shield) throughout the infection. Three biological replicates of infected cells were cross-linked at 24 hpi by adding 16% paraformaldehyde (Electron Microscopy Sciences 15710) to a final concentration of 1% for 10 min at room temperature, followed by incubation with Tris pH 7.6 (final concentration 1.33 M) for 5 min to stop the cross-linking. Cells were washed twice with 5 mL ice-cold PBS, detached from the flask with a scraper, and pelleted at 1200 × *g* for 5 min at 4°C. Pellets were resuspended in 1 mL ice-cold ChIP buffer (10 mM Tris pH 7.6, 150 mM NaCl, 1% Triton X-100, 1 mM EDTA, 0.25% sodium deoxycholate, 1 mM DTT, 0.1% isopropanol-saturated PMSF and cOmplete EDTA-free protease inhibitor cocktail), then sonicated at 20% duty factor, 240 W peak incident power, 200 cycles per burst, 8 min by Covaris E220. The sonicated samples were pelleted at 16000 × *g* for 15 min at 4°C and the supernatants were collected for immunoprecipitations (IP). A T75 flask containing approximately 7.5 × 10^6^ cells was applied to each individual IP. A total of 40 μL Pierce Protein A/G Magnetic Beads (Thermo 88802) were used for each IP performed in triplicate for triplicate infections. A 20 μL volume of these beads were used to preclear the 1 mL of sonicated sample. This entailed washing the beads with 500 μL ChIP buffer, settling the beads with a DynaMag-2 Magnet, and then incubating the beads with the 1 mL of sonicated samples for 2 h at 4°C with rotation. The beads were then removed, and the precleared supernatant was transferred to new tubes. A 10 μL aliquot was removed from precleared sample for use in the 1% input control analysis. The remainder of the precleared sample was incubated with either 10 μg Acetyl-Histone H3 (Lys27) antibody (Cell Signaling D5E4) or 2.5 μg Pol II antibody (Santa Cruz Biotechnology sc-55492) overnight at 4°C with rotation using a HulaMixer Sample Mixer (Invitrogen, 15920D). Before the other 20 μL of magnetic beads were added to the chromatin-antibody samples, they were first washed with 500 μL ChIP buffer, blocked in 500 μL ChIP buffer plus 1 mg/mL BSA overnight at 4°C with rotation, and then washed again with 500 μL ChIP buffer. The blocked magnetic beads were incubated with chromatin-antibody samples for 2 h at 4°C with rotation. Samples were washed five times with 500 μL wash buffer (10 mM Tris pH 7.6, 150 mM NaCl, 1 mM EDTA, 1% Triton X-100, 0.1% sodium deoxycholate and 0.1% SDS), and two times with 500 μL rinse buffer (10 mM Tris pH 7.6, 50 mM NaCl and 1 mM EDTA). Beads were then resuspended in 100 μL elution buffer (10 mM Tris pH 7.6, 1 mM EDTA, and 1% SDS) for 10 min at 65°C. Meanwhile, 90 μL elution buffer was added to the input. To break cross-links and isolate DNA, bead eluates and input control were treated with 2 μL RNase A (Thermo EN0531) for 30 min at 37°C, then incubated with 2 μL Proteinase K (Thermo EO0491) overnight at 65°C. DNA was purified using the MinElute PCR purification kit (Qiagen) in final volume of 100 μL. The amount of each of the indicated DNA target was quantified by qPCR using the oligonucleotide primers listed in Table 1 and PCR buffers and thermocycling parameters described for RT-qPCR. The amount of each specific viral DNA target was determined by standard curve method.

### Data availability.

ChIP-seq and RNA-seq raw data sets and bigwig files are available in GEO superseries GSE171522. A track hub with the HCMV reference genomes has been made available on GitHub for visualization of bigwig files (https://github.com/Mary-Hummel/HCMV-reference-genomes).
