# Supplementary material for: Critical Role for the Human Cytomegalovirus Major Immediate Early Proteins in Recruitment of RNA Polymerase II and H3K27Ac To an Enhancer-Like Element in OriLyt
Source: Microbiol Spectr. 2023 Jan 16;11(1):e03144-22. doi: 10.1128/spectrum.03144-22 (PMC9927211; doi:10.1128/spectrum.03144-22)
Supplement: Supplemental file 1 — Fig. S1 and Table S1. Download spectrum.03144-22-s0001.pdf, PDF file, 3.5 MB [file spectrum.03144-22-s0001.pdf]

Supplemental material for publication

Critical Role for the Human Cytomegalovirus major immediate early proteins in recruitment of RNA polymerase II and H3K27Ac to an enhancer-like element in Ori*Lyt*

Eleonora Forte[1,2*§], Ming Li[3§], Fatma Ayaloglu Butun[1,2], Qiaolin Hu[3], Eva Maria Borst[4], Matthew J. Schipma[5], Andrea Piunti[6†], Ali Shilatifard[6], Scott S. Terhune[7], Michael Abecassis[1£], Jeffery L. Meier[#3] and Mary Hummel[#¶1]

[1] Comprehensive Transplant Center, Department of Surgery, Northwestern University Feinberg School of Medicine, Chicago, IL, USA

[2] Proteomics Center of Excellence, Northwestern University, Evanston, IL

[3]Departments of Internal Medicine and Epidemiology, University of Iowa and Iowa City Veterans Affairs Health Care System, Iowa City, Iowa

[4]Department of Virology, Hannover Medical School, Hannover, Germany

[5]NUSeq Core, Quantitative Data Science Core, Northwestern University Feinberg School of Medicine, Chicago, IL, USA

[6]Department of Biochemistry and Molecular Genetics, Northwestern University Feinberg School of Medicine, Chicago, IL, USA

[7] Department of Microbiology and Immunology and Biotechnology and Bioengineering Center, Medical College of Wisconsin, Milwaukee, WI, USA

[†]Current address: Department of Pediatrics, University of Chicago, Chicago, IL
[£]Current address: College of Medicine-Tucson, University of Arizona Health Sciences, Tucson, AZ

*Corresponding author: e-forte@northwestern.edu, 2170 Campus Drive, Silverman Hall 3710, Evanston, IL 60208
[§]These authors contributed equally to the work.
[#]Co-senior authors
[¶]retired

Contents

Supplemental material legends
Figure S1
Table S1

Supplemental material legends

Fig. S1. Enlarged view of the landscape of the HCMV transcriptome and epigenomes. Bigwig tracks of RNAseq, Pol II ChIPseq, and H3K27Ac ChIPseq aligned to TB40/E*wt*-GFP genome are shown in sections of ~24kb. Note that the scales differ in each panel to enhance visualization of the peaks in each region. The top of each panel shows the coordinates of the genome. The bottom of each panel shows the open reading frames (ORFs). Not all ORFs are shown.

Table S1. Log2 of HCMV read counts in TB40/E*wt*-GFP -infected MRC5 cells at 24 hpi.

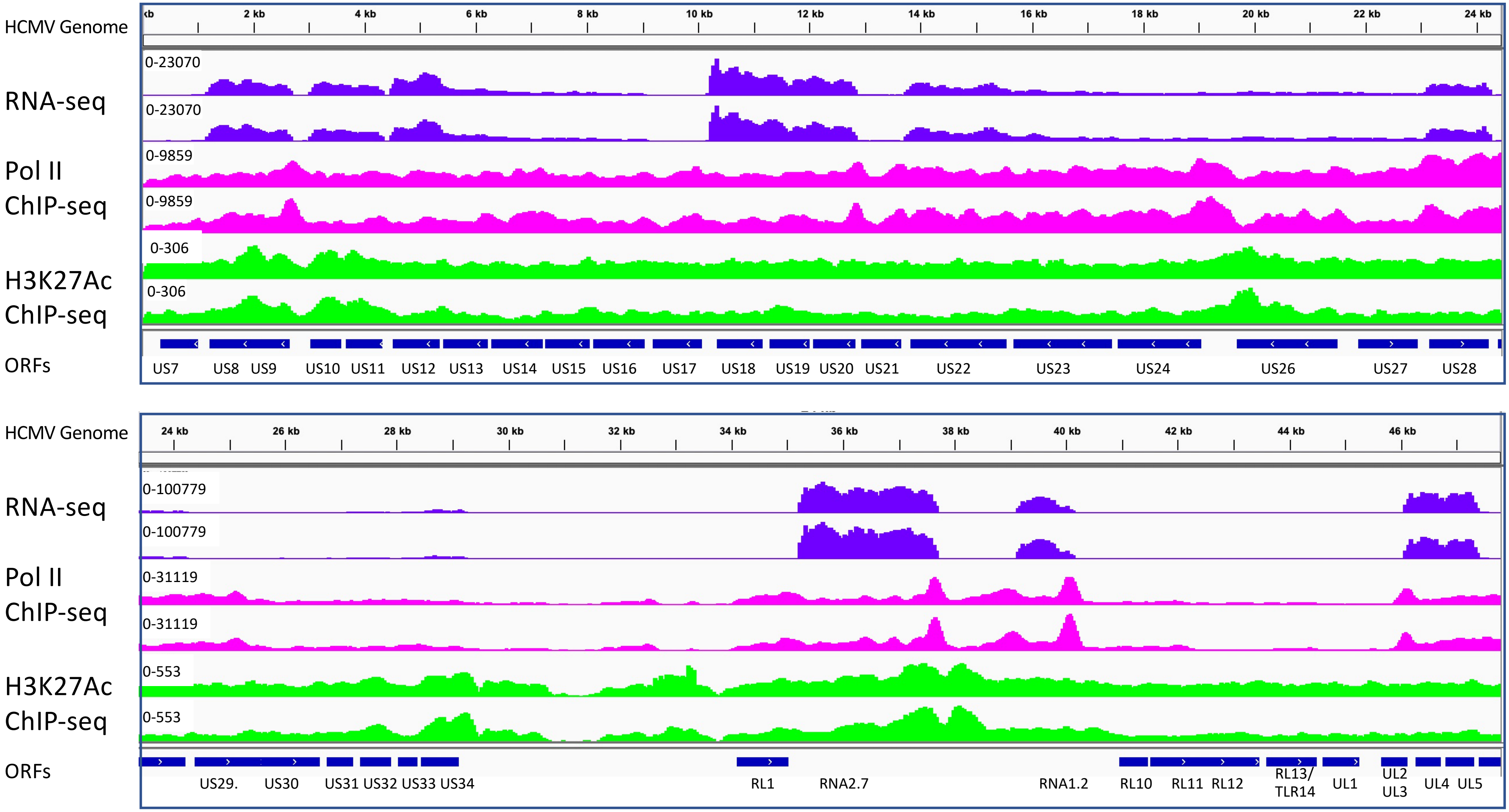

HCMV Genome
RNA-seq
Pol II
ChIP-seq
H3K27Ac
ChIP-seq
ORFs
2 kb
4 kb
6 kb
8 kb
10 kb
12 kb
14 kb
16 kb
18 kb
20 kb
22 kb
24 kb
0-23070
0-23070
0-9859
0-9859
0-306
0-306
US7
US8
US9
US10
US11
US12
US13
US14
US15
US16
US17
US18
US19
US20
US21
US22
US23
US24
US26
US27
US28
HCMV Genome
RNA-seq
Pol II
ChIP-seq
H3K27Ac
ChIP-seq
ORFs
24 kb
26 kb
28 kb
30 kb
32 kb
34 kb
36 kb
38 kb
40 kb
42 kb
44 kb
46 kb
0-100779
0-100779
0-31119
0-31119
0-553
0-553
US29.
US30
US31
US32
US33
US34
RL1
RNA2.7
RNA1.2
RL10
RL11
RL12
RL13/
TLR14
UL1
UL2
UL3
UL4
UL5

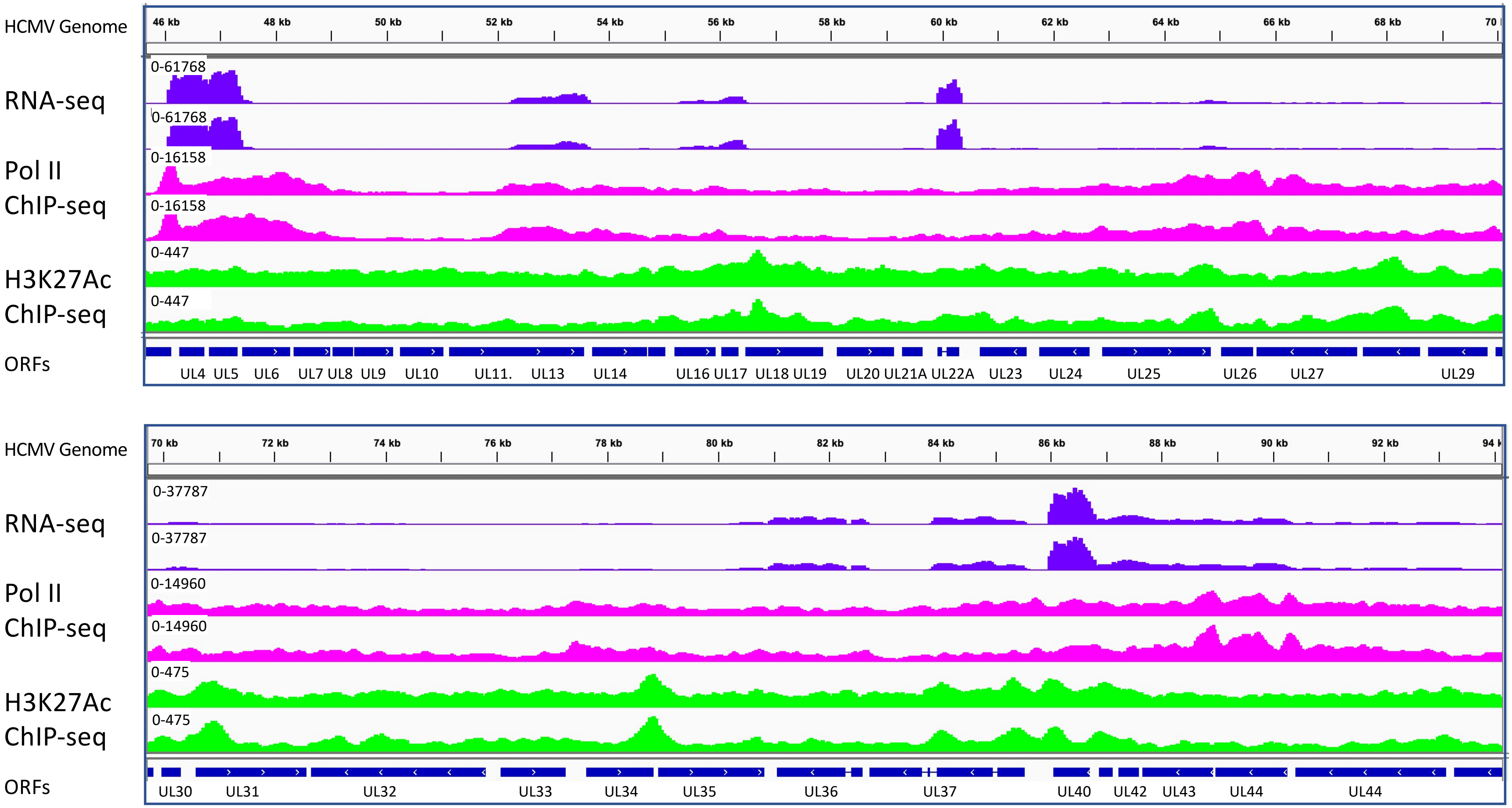
HCMV Genome
46 kb
48 kb
50 kb
52 kb
54 kb
56 kb
58 kb
60 kb
62 kb
64 kb
66 kb
68 kb
70
RNA-seq
0-61768
0-61768
Pol II
ChIP-seq
0-16158
0-16158
H3K27Ac
ChIP-seq
0-447
0-447
ORFs
UL4 UL5 UL6 UL7 UL8 UL9 UL10 UL11. UL13 UL14 UL16 UL17 UL18 UL19 UL20 UL21A UL22A UL23 UL24 UL25 UL26 UL27 UL29
HCMV Genome
70 kb
72 kb
74 kb
76 kb
78 kb
80 kb
82 kb
84 kb
86 kb
88 kb
90 kb
92 kb
94
RNA-seq
0-37787
0-37787
Pol II
ChIP-seq
0-14960
0-14960
H3K27Ac
ChIP-seq
0-475
0-475
ORFs
UL30 UL31 UL32 UL33 UL34 UL35 UL36 UL37 UL40 UL42 UL43 UL44 UL44

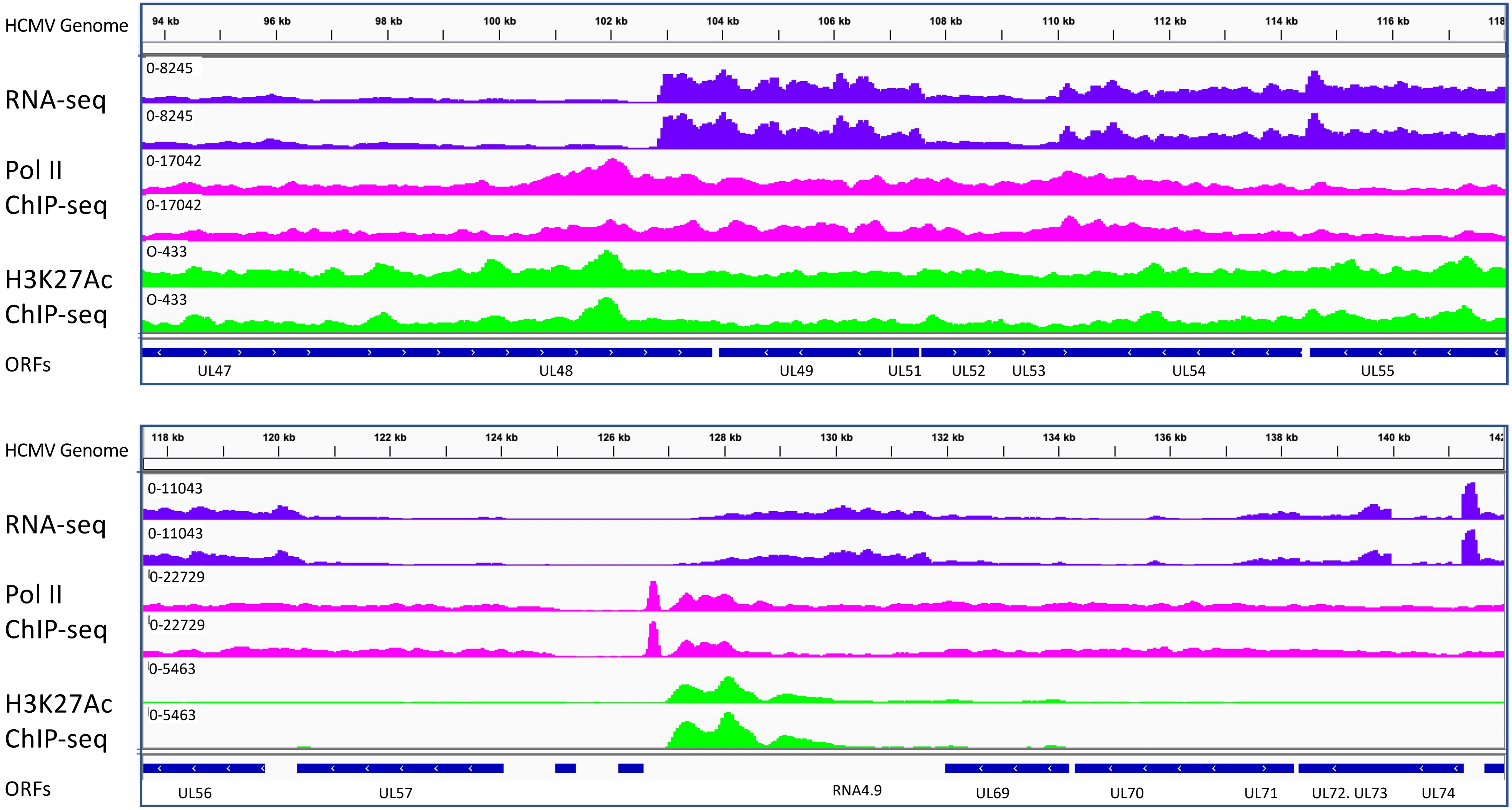

HCMV Genome
94 kb
96 kb
98 kb
100 kb
102 kb
104 kb
106 kb
108 kb
110 kb
112 kb
114 kb
116 kb
118
RNA-seq
0-8245
0-8245
Pol II ChIP-seq
0-17042
0-17042
H3K27Ac ChIP-seq
O-433
O-433
ORFs
UL47
UL48
UL49
UL51
UL52
UL53
UL54
UL55
HCMV Genome
118 kb
120 kb
122 kb
124 kb
126 kb
128 kb
130 kb
132 kb
134 kb
136 kb
138 kb
140 kb
RNA-seq
0-11043
0-11043
Pol II ChIP-seq
0-22729
0-22729
H3K27Ac ChIP-seq
0-5463
0-5463
ORFs
UL56
UL57
RNA4.9
UL69
UL70
UL71
UL72. UL73
UL74

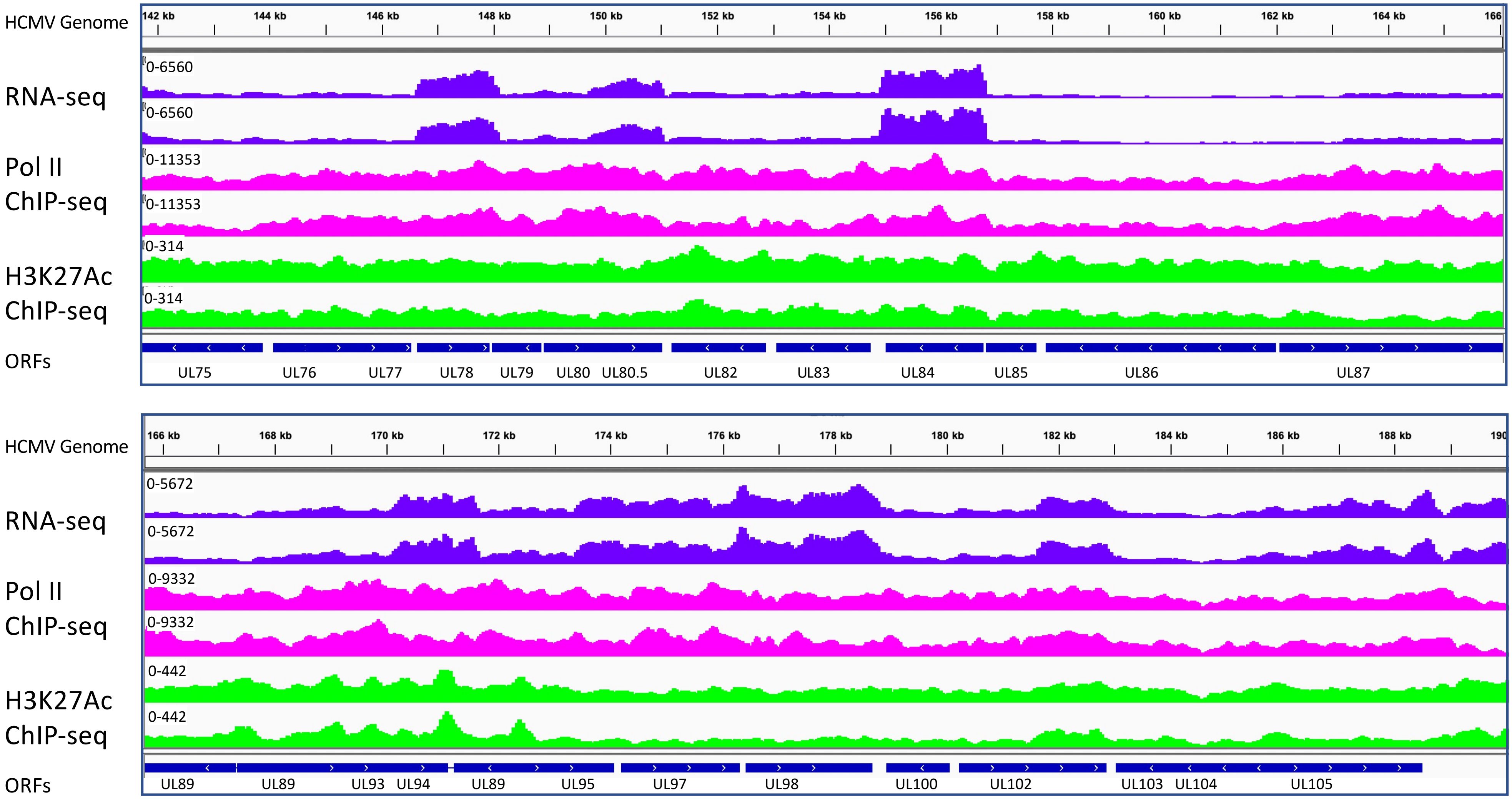
HCMV Genome
142 kb
144 kb
146 kb
148 kb
150 kb
152 kb
154 kb
156 kb
158 kb
160 kb
162 kb
164 kb
166
RNA-seq
0-6560
0-6560
Pol II
ChIP-seq
0-11353
0-11353
H3K27Ac
ChIP-seq
0-314
0-314
ORFs
UL75
UL76
UL77
UL78
UL79
UL80
UL80.5
UL82
UL83
UL84
UL85
UL86
UL87
HCMV Genome
166 kb
168 kb
170 kb
172 kb
174 kb
176 kb
178 kb
180 kb
182 kb
184 kb
186 kb
188 kb
190
RNA-seq
0-5672
0-5672
Pol II
ChIP-seq
0-9332
0-9332
H3K27Ac
ChIP-seq
0-442
0-442
ORFs
UL89
UL89
UL93
UL94
UL89
UL95
UL97
UL98
UL100
UL102
UL103
UL104
UL105

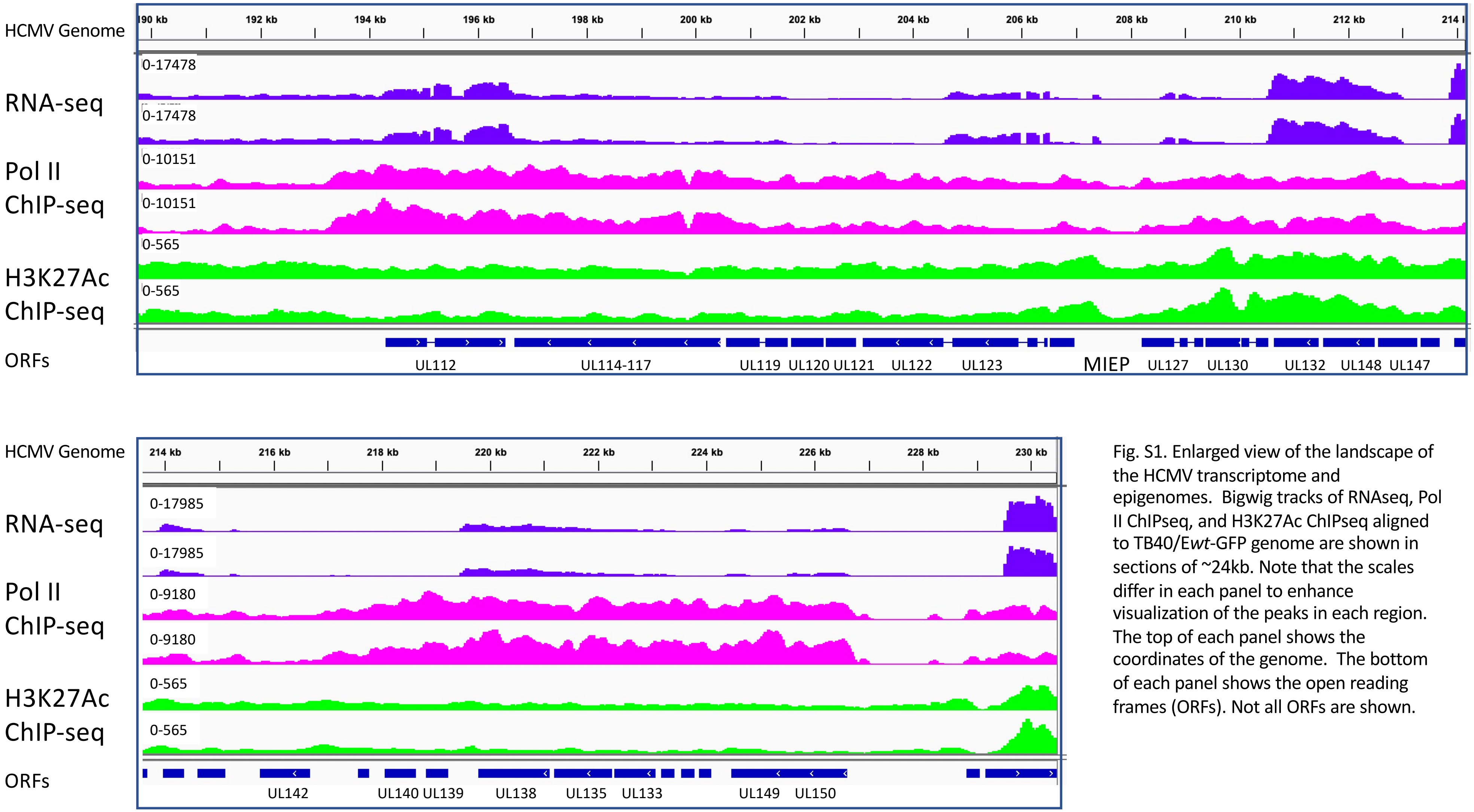

Fig. S1. Enlarged view of the landscape of the HCMV transcriptome and epigenomes. Bigwig tracks of RNAseq, Pol II ChIPseq, and H3K27Ac ChIPseq aligned to TB40/E*wt*-GFP genome are shown in sections of ~24kb. Note that the scales differ in each panel to enhance visualization of the peaks in each region. The top of each panel shows the coordinates of the genome. The bottom of each panel shows the open reading frames (ORFs). Not all ORFs are shown.

Table S1. Log2 of HCMV read counts in TB40/Ewt-GFP -infected MRC5 cells

Gene	Sample_1	Sample_2
GFP	17.505548	17.3548692
RL1	8.73470962	9.10066234
RL10	9.15987134	9.42626475
RL11	10.6943579	10.9329529
RL12	12.6786001	12.9025635
RL13/TRL14	12.3303567	12.6950107
UL1	8.94251451	9.15987134
UL10	10.0443941	10.258566
UL100	12.0640691	12.1169937
UL102	14.1112987	14.2352663
UL103	10.9299981	10.909143
UL104	11.8765169	11.9512847
UL105	14.1710986	14.1781202
UL11	10.4335854	10.5689062
UL112	15.3554199	15.5178846
UL114	12.2905949	12.3261486
UL115	12.1186167	12.0794848
UL116	11.9654236	11.9414145
UL117	12.2615073	12.2839565
UL119	12.2914585	12.3509392
UL12	7.80735492	8.08746284
UL120	9.55842071	9.54689446
UL121	10.1636497	10.0593445
UL122	11.4387919	11.590587
UL123	13.5623617	14.0520585
UL124	10.2691267	10.4918531
UL127	4.64385619	5.04439412
UL128	11.6763979	11.8169836
UL13	15.8410237	15.4377846
UL130	10.6447576	10.8033239
UL131A	9.6635581	9.87498135
UL132	14.8551605	14.9242558
UL133	13.479654	13.4290144
UL134	5.20945337	4.80735492
UL135	14.3755146	14.2840288
UL136	14.3067737	14.326921
UL137	3.169925	2.80735492
UL138	14.3496959	14.4610957
UL139	10.5087852	10.6608873

UL14	11.7274951	11.8249587
UL140	11.3106128	11.5018372
UL142	9.22881869	9.40301202
UL144	12.7473538	12.9434306
UL145	14.4471478	14.2172762
UL146	8.34429591	8.53527538
UL147	10.203348	10.2419831
UL147A	9.99859043	10.1344263
UL148	14.5097132	14.5296746
UL148A	11.6821168	11.7402024
UL148B	10.0209799	10.1799091
UL148C	10.6882503	10.911392
UL148D	9.96000193	9.98013958
UL150	14.4675419	14.5857254
UL15A	10.1799091	10.2946207
UL16	13.4995968	13.533208
UL17	13.7731392	14.0088663
UL18	9.69348696	9.82017896
UL19	9.65105169	9.96289601
UL2	7.26678654	7.90086681
UL20	12.1702381	12.2737956
UL21A	11.7444134	11.8494051
UL22A	15.209796	15.5437569
UL23	12.2401951	12.5427901
UL24	12.3286749	12.4797803
UL25	14.0916833	14.2700763
UL26	12.6434053	12.742941
UL27	13.4628856	13.6246812
UL29	12.5230718	12.7825888
UL3	13.8087631	13.6773893
UL30	11.9314762	12.2354159
UL31	13.436191	13.6417131
UL32	13.335809	13.5409756
UL33	11.7270696	11.7817695
UL34	12.580023	12.6849677
UL35	12.8311093	12.9307373
UL36	15.1601078	15.0532896
UL37	11.2997804	11.4817994
UL38	14.6628909	14.7158548
UL4	16.2727756	16.3127943
UL40	16.2466478	16.1191032

UL41A	12.5233169	12.6926155
UL42	13.4252159	13.5160693
UL43	14.3686429	14.499223
UL44	15.4400581	15.6107077
UL46	12.1360298	12.3049217
UL47	13.5507468	13.7244073
UL48	15.0660051	15.2349297
UL48A	11.6978364	11.7223806
UL49	14.3199543	14.3756504
UL5	16.4285074	16.3951492
UL50	13.8428414	13.9864643
UL51	12.0052737	11.9578276
UL52	13.2306209	13.2149262
UL53	12.6757366	12.8039293
UL54	15.1971783	15.2620948
UL55	15.1235152	15.186269
UL56	14.528271	14.6281037
UL57	13.2002856	13.3027817
UL59	4.24792751	4.45943162
UL6	12.3605728	12.4452736
UL60	4.64385619	5.28540222
UL69	12.8267471	13.0110525
UL7	9.37503943	9.74819285
UL70	12.0147179	12.1721149
UL71	12.5973543	12.6646695
UL72	12.1962947	12.2407913
UL73	11.2544381	11.1337844
UL74	11.9140113	11.9087679
UL75	13.0117514	13.022021
UL76	10.1382718	10.1006623
UL77	11.7146748	11.7854525
UL78	13.9803181	13.927778
UL79	10.987264	10.9893945
UL8	8.37503943	8.77478706
UL80	11.5038257	11.5507468
UL82	12.4437206	12.6325409
UL83	12.5154535	12.6982706
UL84	14.7652861	14.9980174
UL85	10.8360504	10.9679467
UL86	12.4333246	12.4900988
UL87	12.57483	12.8013042

UL88	11.4665863	11.6821168
UL89	12.8950072	13.1310531
UL9	9.49984589	9.88264305
UL90	5.39231742	5.93073734
UL91	7.93073734	7.9068906
UL92	10.243174	10.4304526
UL93	12.6377569	12.8501868
UL94	13.0377185	13.202736
UL95	12.7929936	13.064406
UL96	11.5764843	11.6943579
UL97	14.1519679	14.3052068
UL98	14.0373754	14.2098341
UL99	12.8239643	12.8780509
US10	13.689998	13.6743026
US11	13.6028148	13.5824948
US12	14.945581	14.8034248
US13	13.3618067	13.2911707
US14	12.8006977	12.762382
US15	12.3788367	12.3545249
US16	12.3462367	12.3820836
US17	10.8879821	11.095397
US18	15.5715157	15.5424273
US19	14.5626598	14.5145291
US20	14.3974072	14.3918477
US21	10.9794252	11.1150437
US22	15.1801034	15.2734314
US23	14.0059754	14.1325801
US24	13.2785946	13.4099212
US26	13.9020924	14.100416
US27	12.9100805	13.1145557
US28	14.6152273	14.7552527
US29	11.7519624	11.795228
US30	12.5186532	12.6094096
US31	12.3581017	12.3885555
US32	12.6368512	12.8035257
US33	11.911392	11.7380923
US34	13.4482454	13.366049
US34A	12.2755426	12.1623913
US7	10.592457	10.6082548
US8	13.991699	14.0555366
US9	13.9465408	13.9202598
